# Profilin 1 Induces Tumor Metastasis by Promoting Microvesicle Secretion Through the ROCK 1/p-MLC Pathway in Non-Small Cell Lung Cancer

**DOI:** 10.3389/fphar.2022.890891

**Published:** 2022-05-02

**Authors:** Ya Wang, Yichen Lu, Rongjun Wan, Yang Wang, Chunfang Zhang, Min Li, Pengbo Deng, Liming Cao, Chengping Hu

**Affiliations:** ^1^ Department of Respiratory Medicine, Xiangya Hospital, Central South University, Changsha, China; ^2^ Department of Oncology, Hunan Provincial People’s Hospital/The First Affiliated Hospital of Hunan Normal University, Changsha, China; ^3^ Department of Thoracic Surgery, Xiangya Hospital, Central South University, Changsha, China

**Keywords:** non-small cell lung cancer, Profilin 1, microvesicles, ROCK/p-MLC pathway, metastasis

## Abstract

Profilin 1 (PFN1), an actin-binding protein, plays contrasting roles in the metastasis of several cancers; however, its role in non-small cell lung cancer (NSCLC) metastasis remains unclear. Here, PFN1 expression was upregulated in metastatic NSCLC tissues. PFN1 overexpression significantly promotes NSCLC metastasis *in vitro* and *in vivo*. Proteomics analysis revealed PFN1 involvment in microvesicles (MVs) secretion. *In vitro* experiments confirmed that PFN1 overexpression increased secretion of MVs. MVs are important mediators of metastasis. Here, we show an increased abundance of MVs in the sera of patients with metastatic NSCLC compared to that in the sera of patients with non-metastatic NSCLC. Both *in vitro* and *in vivo* experiments revealed that PFN1 could increase MV secretion, and MVs derived from PFN1-overexpressing cells markedly promoted NSCLC metastasis. We then elucidated the mechanisms underlying PFN1-mediated regulation of MVs and found that PFN1 could interact with ROCK1 and enhance its kinase activity to promote myosin light chain (MLC) phosphorylation for MV secretion. Inhibition of ROCK1 decreased MV secretion and partially reversed the PFN1-induced promotion of NSCLC metastasis. Collectively, these findings show that PFN1 regulates MV secretion to promote NSCLC metastasis. PFN1 and MVs represent potential predictors or therapeutic targets for NSCLC metastasis.

## Introduction

Non-small cell lung cancer (NSCLC) accounts for ∼85% of all lung cancer cases and has a poor 5-year survival rate of ∼15% (Chen et al., 2014; Siegel et al., 2018). Despite major advances in treatment, the prognosis of lung cancer remains poor owing to extensive metastasis at diagnosis (Herbst et al., 2018). Lung cancer cells metastasize to distant organs through a complex process called the invasion–metastasis cascade (Fidler, 2003; Gupta and Massagué, 2006; Talmadge and Fidler, 2010), during which molecular signal exchange between tumor cells and between tumor cells and the stroma is crucial (Dudas, 2015).

Profilin 1 (PFN1) is a 135-amino acid, 15 kDa conservative actin-binding protein. PFN1 is expressed at every stage of embryonic development and is ubiquitously expressed by all cell types and nearly all tissues (Alkam et al., 2017). Numerous studies have confirmed that PFN1 involved in tumor proliferation, apoptosis, stemness, immune response and metastasis (Liao et al., 2021; Wang et al., 2021). Specifically, large amount of studies focused on its roles in cancer metatasis. Accumulating evidence suggests that PFN1 participates in the metastasis of breast cancer (Ding et al., 2014), hepatocellular carcinoma (Wang et al., 2019), and renal cell cancer (Karamchandani et al., 2015). However, the role of PFN1 differs from cancer to cancer, and the mechanisms underlying PFN1 function in cancer metastasis are not fully understood. Numerous studies revealed that PFN1 plays a vital role in membrane trafficking (Dong et al., 2000; Valenzuela-Iglesias et al., 2015; Lu et al., 2018), thus providing insights into the mechanism underlying its function in cancer metastasis.

Extracellular vesicles are a heterogeneous group of nano-scale lipid-bilayered structures secreted by all cell types, including cancer cells. They are classified by size as exosomes (40–120 nm), microvesicles (MVs; 50–1,000 nm), or apoptotic bodies (500–2,000 nm) (EL Andaloussi et al., 2013; Liang et al., 2021) and play a critical role in cell-to-cell communication (Balaj et al., 2011; Chiodoni et al., 2019; Lv et al., 2019). Tumor cells can release large quantities of MVs, the abundance of which correlates with tumor invasiveness and development (Muralidharan-Chari et al., 2010; Menck et al., 2020). Proteins, lipids, and nucleic acids, including various microRNAs (miRNAs) and long non-coding RNAs (lncRNAs), are selectively trafficked into MVs, where they ultimately affect gene expression regulation and cell functions (Raposo and Stoorvogel, 2013; Tricarico et al., 2017; Fabbiano et al., 2020). Unlike exosomes, MVs bud directly from the cell membrane (Tricarico et al., 2017), a process that relies on actin-myosin interactions and ATP-dependent contraction. Phosphorylation of myosin light chain (MLC) at the neck of the budding vesicle is mediated by either the rho-associated coiled-coil containing kinase (ROCK) signaling pathway (Li et al., 2012) or the ADP-ribosylation factor 6 (ARF6) (Muralidharan-Chari et al., 2009), which activate MLC and promote MV release. As an important actin-binding protein, PFN1 is closely related to cytoskeletal regulation and membrane trafficking. However, its role in MV secretion is elusive, and whether PFN1 affects NSCLC metastasis by regulating MV secretion is unclear.

In this study, we systematically explored the roles of PFN1 in tumor metastasis and MV secretion in NSCLC, and confirmed that PFN1 promoted NSCLC metastasis by promoting the secretion of MVs *in vitro* and *in vivo*. Mechanistically, we demonstrated that PFN1 interacted with ROCK1, followed by its activation, and thus promoted phosphorylation of MLC, which in turn increased the secretion of MVs.

## Materials and Methods

### Patients and Ethics Statement

Forty-five patients with lung adenocarcinoma admitted to our hospital were divided into metastatic and non-metastatic groups, according to the 2019 Chinese Society of Clinical Oncology guidelines (Supplementary Table S1). Patients with definite pathological diagnosis of lung adenocarcinoma and available clinical information were included. Patients with complications of other malignant diseases, inflammatory diseases, or chronic diseases, such as diabetes, hypertension, or coronary heart diseases, were excluded before recruiment. The study design and protocol were approved by the Ethics Committee of Hunan Provincial People’s Hospital/The First Affiliated Hospital of Hunan Normal University (Changsha, Hunan, China, 2020-Provincial 02) and adhered to the ethical guidelines of the Declaration of Helsinki. Informed consent was obtained from all participants.

### Tissue Chip and Immunohistochemistry

Tissue chips were purchased from Shanghai Outdo Biotech (Shanghai, China) (Supplementary Table S2). For animal experiments, lung tissues were harvested and fixed in 4% paraformaldehyde. The fixed samples were embedded in paraffin; 4-µm-thick sections were cut onto glass slides, and immunohistochemistry analysis was conducted as described previously (Feng et al., 2020). Before staining, the slides were heated at 60°C and then treated with an alcohol gradient. After removing endogenous catalase with 3% hydrogen peroxide, the slides were blocked with 3% normal sheep serum (ZSbio, Beijing, China) at room temperature for 1 h. The samples were then incubated overnight with primary antibodies at 4°C. Primary antibody concentrations were as follows: anti-PFN1, 1:500 (Abcam, Cambridge, MA, United States) and anti-phosphorylated (p)-MLC, 1:100 (Abclonal, Wuhan, China, phosphorylation site is at S18) (catalog numbers listed in Supplementary Table S3). Protein expression was determined using the VECTASTAIN Elite ABC HRP Kit (Vector Laboratories, Burlingame, CA, United States) and VECTOR DAB Kit (Vector Laboratories) following the manufacturer’s instructions. Images were acquired using a PANNORAMIC whole slide scanner (3DHISTECH, Budapest, Hungary). The staining intensity was the product of staining characteristics of the target cell (no staining was scored as 0, light yellow as 1, yellow/brown as 2, and brown as 3) and the positive rate of cells (0–5% was scored as 0, 6–25% as 1, 26–50% was 2, 51–75% as 3, and >75% as 4).

### Immunofluorescence

Tissues were fixed with 4% paraformaldehyde and permeabilized with 0.2% Triton X-100. After blocking with 3% bovine serum albumin (BSA; Servicebio, Wuhan, China), the slides were incubated with primary antibodies overnight at 4°C. Primary antibody concentrations were as follows: anti-PFN1, 1:100 (Abcam), anti-Napsin A, 1:100 (Abcam), anti-ROCK1, 1:100, (ImmunoWay, Plano, TX, United States), anti-ROCK2, 1:100 (ImmunoWay) and anti-annexin A1, 1:100 (Servicebio). Next, the slides were incubated with fluorophore-conjugated secondary antibodies (1:5,000, Proteintech, Wuhan, China) at room temperature. DAPI (Servicebio) was used to stain the nuclei. The samples were then visualized using a fluorescence microscope (Nikon, Tokyo, Japan).

### Cell Culture and Treatment

H1299 and A549 cell lines were kindly provided by Stem Cell Bank, Chinese Academy of Sciences (Beijing, China). HEK-293T cells were kindly provided by Professor Chun Fang Zhang (Department of Thoracic Surgery, Xiangya Hospital, Central South University). Cells were cultured in RPMI 1640 medium (Gibco, Waltham, MA, United States) or Dulbecco’s modified Eagle medium (Gibco) (HEK-293T cells only) supplemented with 10% fetal bovine serum (Gibco), 100 U/mL penicillin, and 100 μg/ml streptomycin (Gibco) and incubated at 37°C and 5% CO_2_.

### Plasmid Construction and Transfection

The human PFN1-coding sequence was amplified from human cDNA using 2× Phanta Master Mix (Vazyme, NanJing, China) and cloned into a pLVX-IRES-ZsGreen1 and pEGFP-C3 vector using the ClonExpress II One Step Cloning Kit (Vazyme). The primers were synthesized by TsingKe Biology Company (Beijing, China) and are listed in Supplementary Table S4. After gene recombination and transformation into *Escherichia coli*, plasmids were extracted using the FastPure Plasmid Mini Kit (Vazyme). The plasmids were then selected according to sequence screening. All genes were transfected into cells using lentivirus infection. pLVX-EF1α-IRES-puro, pLVX-EF1α-IRES-puro-PFN1-Flag, pLVX-EF1α-IRES-puro-PFN1-R88L-Flag, pLVX-EF1α-IRES-puro-PFN1-H119E-Flag, pLVX-EF1α-IRES-puro-PFN1-H133S-Flag, and pLVX-EF1α-IRES-puro-ROCK2-myc were purchased from General Biology Company (Chuzhou, China). ROCK1 pcDNA3.1-HA-C was purchased from You Bao Biology (Changsha, China). PFN1 siRNA and control scramble siRNA were synthesized by Guangzhou RiboBio Co. (Guangzhou, China), and the sequences are listed in Supplementary Table S4. Plasmids and siRNAs were transfected into cells using Lipofectamine™ 3000 Transfection Reagent (Invitrogen, Waltham, MA, United States) according to the manufacturer’s instructions.

### Wound Healing Assay

Cells in the logarithmic growth phase were seeded onto six-well plates (3 × 10^5^ cells/well). The supernatants were collected from cultured cells transfected with the empty vector (EV) and those overexpressing PFN1. Pipette tips were used to make a straight scratch in each well; we ensured that the width of each scratch was similar. Images were captured under microscopy (Nikon) every 12 h until the wound was healed. Data were obtained from at least three independent experiments.

### Transwell Migration Assay

Cell migration was assessed using 24-well Transwell culture chambers (Corning, Corning, NY, United States). Cells (2 × 10^4^) suspended in 200 µl serum-free medium were seeded in the upper chamber. The lower chamber was filled with 800 µl complete medium. After incubation at 37°C for 24 h, the cells were fixed in 4% paraformaldehyde for 20 min. After being washed thrice with PBS, the cells were stained using 1% crystal violet (Solarbio, Beijing, China) for 10 min. Cells that transmigrated to the lower chamber were counted. Data were obtained from at least three independent experiments.

### Isolation of Microvesicles

Microvesicles (MVs) were isolated using continuous differential centrifugation as described previously (Jeppesen et al., 2019). Briefly, H1299 and A549 cells were divided into two groups: EV and PFN1 overexpression. Approximately 2.5 × 10^6^ cells at log phase were seeded into T75 cell culture flasks with serum-free medium. After being incubated at 37°C for 48 h, cell supernatants were collected. The supernatants were centrifuged at 750 × *g* for 5 min followed by centrifugation at 1,500 × *g* for 10 min to remove cell debris. The subsequent supernatants were centrifuged at 16,000 × *g* for 45 min. The obtained pellets were resuspended in 1 ml PBS and centrifuged at 16,000 × *g* for 45 min. A total of 40 µl PBS was used to resuspend the pellets for subsequent studies. MVs in the serum were also extracted using continuous differential centrifugation and stored at −80°C for use in subsequent analyses.

### Electron Microscopy

MVs were fixed in 2.5% glutaraldehyde. Electron microscopy was conducted at the Department of Pathology of Xiangya Hospital, Central South University (Changsha, China).

### Flow Cytometry for Quantitative Analysis of Microvesicles

The amount of MVs was analyzed using a three-laser Cytek Northern Lights SpectralFlow Cytometry instrument (Cytek, Fremont, CA, United States), according to the manufacturer’s instructions. Standard microbeads (ranging from 100 nm to 1 µm) were used for size calibration. Microparticles ranging from 100 nm to 1 µm were counted. The counting endpoint was set at 25 s for all eligible granules.

### Detection of Microvesicle Uptake

MVs stained with PKH67 (Sigma, Darmstadt, Germany) were added to H1299 cells. After incubation for 24 h, cells were fixed and stained with DAPI. Then, fluorescence microscopy (Nikon) was used to detect MS uptake.

### RNA Extraction and Reverse Transcription-Quantitative PCR

Total RNA was extracted from cells at the logarithmic phase using TRIzol reagent (Takara, Kyoto, Japan). Reverse transcription was conducted using the PrimeScript RT Reagent Kit (Takara). RT-qPCR was conducted on a 96-well Automation Compatible Polypropylene PCR Microplate (Axygen, Darmstadt, Germany) using the TB Green Premix qPCR Mix (Takara) per manufacturer’s instructions. Primer sequences are listed in Supplementary Table S4. Data were obtained from at least three independent experiments.

### Protein Extraction, Western Blotting, and Co-Immunoprecipitation

Whole-cell lysates were prepared using RIPA Lysis Buffer (Beyotime, Shanghai, China) containing a protease inhibitor cocktail (Bimake, Houston, TX, United States) and phosphatase inhibitor (Roche, Basel, Switzerland). The protein concentration was quantified using a BCA Kit (Beyotime). An equal amount of protein (25 µg) was used for electrophoresis. Proteins were separated using 10/12% SDS PAGE and then transferred onto polyvinylidene difluoride membranes (Millipore, Billerica, MA, United States). The membranes were then blocked with a solution of 3% BSA (Solarbio) in TBS with Tween 20 (Solarbio) at room temperature for 1 h. The membranes were next incubated with primary antibodies (dilution ratio listed in Supplementary Table S3) overnight at 4°C, followed by incubation with horseradish peroxidase-conjugated anti-rabbit or anti-mouse secondary antibodies (1:5,000, Proteintech) at room temperature for 2 h. After washing, the protein bands were detected using Luminata Western HRP Substrate (Millipore) and a Gene Genius Bioimaging System (Bio-Rad, Hercules, CA, United States). Co-IP was conducted using Pierce™ Protein A/G Magnetic Beads (Thermo Fisher Scientific) per manufacturer’s instructions. Data were obtained from at least three independent experiments.

### ROCK Kinase Assay

Purified ROCK1/2, PFN1, and PFN1 mutants were isolated from HEK-293T cells transfected with *ROCK1*/*2* or *PFN1* overexpression/mutant plasmids using Pierce™ Protein A/G Magnetic Beads (Thermo Fischer Scientific) per manufacturer’s instruction. The ROCK Kinase assay (Abcam) was used to evaluate the effect of PFN1 or its mutants on ROCK1/2 activity per the manufacturer’s instructions. Data were obtained from at least three independent experiments.

### *In Vivo* Metastasis Assay

Nude mice (4 weeks old, female, 18–20 g) were purchased from Hunan SJA Laboratory Animal Co., Ltd. (Changsha, China). The mice were nurtured in individual ventilated caging systems under a specific pathogen-free environment. A metastasis model was constructed via intracardiac injection of H1299 cells. Briefly, after being anesthetized by injecting 2% sodium pentobarbital (40 mg/kg) intraperitoneally, the mice’s skin was cut along the left parasternal line to locate the second intercostal space. Needles were inserted into the second intercostal space along the left margin of the sternum. Pulsating arterial blood indicated the correct location in the left ventricle. A total of 8 × 10^5^ H1299 cells/mouse, suspended in PBS, were injected immediately. MVs (tatol MVs collected from 3 × 10^6^ H1299 EV cells or PFN1 OE cells for each mouse) were injected together with cells. When significant weight loss was observed, the mice were sacrificed for subsequent analyses. All animal experimental procedures were performed in accordance with the Guide for the Care and Use of Laboratory Animals of Hunan Provincial People’s Hospital/The First Affiliated Hospital of Hunan Normal University and approved by the Institutional Animal Ethics Committee. All animal experiments complied with the ARRIVE guidelines.

### Sample Preparation for Proteomics

H1299 EV and PFN1 OE cells were harvested at logarithmic growth period and each cell type was collected in biological triplicate. Total cellular protein extracted by RIPA lysis buffer and concentration of proteins were determined by bicinchoninic acid (BCA) protein assay kit (Beyotime) according to manufacturer instructions. A total of 100ug proteins of each sample were then precipitated by acetone at −20°C overnight. Dissolve protein precipitation by water bath ultrasound for 3 min after adding 100 µl protein resolve buffer. Dithiothreitol (DTT) (Sigma-Aldrich) was used to reduced disulfide bond. Then reduced disulfide bonds were Alkylated by iodoacetamide (IAA) (Sigma-Aldrich). Thoroughly mixed trypsin with the samples in the ratio of trypsin: protein = 1:50 and incubate overnight at 37°C 1,000 rpm to digest proteins. Peptides were labeled by TMT Isobaric Label Reagent Set (Thermo Fisher Scientific, Waltham, MA, United States) according to manufacturer instructions. After SDS cleanup, peptide desalting and gigh-pH pre-fractionation, peptides were freeze at 80°C after vacuum drying and ready for later nanoLC-MS/MS analysis.

### NanoLC-MS/MS Analysis

For each sample, 2 ug of total peptides were separated and analyzed with a nano-UPLC (EASY-nLC1200) coupled to a Q Exactive HFX Orbitrap instrument (Thermo Fisher Scientific) with a nano-electrospray ion source. Separation was performed by using a reversed-phase column (100 *µ* ID ×15 cm, Reprosil-Pur 120 C18-AQ, 1.9 *µ*, Dr. Maisch). Mobile phases were H_2_O with 0.1% FA, 2% ACN (phase A) and 80% ACN, 0.1% FA (phase B). Separation of sample was executed with a 90 min gradient at 300 nl/min flow rate. Gradient B: 2–5% for 2 min, 5–22% for 68 min, 22–45% for 16 min, 45–95% for 2 min, 95% for 2 min.

Data dependent acquisition (DDA) was performed in profile and positive mode with Orbitrap analyzer at a resolution of 120,000 (@200 m/z) and m/z range of 350–1,600 for MS1. For MS2, the resolution was set to 15k with a fixed first mass of 110 m/z. The automatic gain control (AGC) target for MS1 was set to 3E6 with max IT 30 ms, and 1E5 for MS2 with max IT 96 ms. The top 20 most intense ions were fragmented by HCD with normalized collision energy (NCE) of 32%, and isolation window of 0.7 m/z. The dynamic exclusion time window was 45 s, single charged peaks and peaks with charge exceeding 6 were excluded from the DDA procedure.

### MS Data Analysis

Raw MS files were processed using Proteome Discoverer (PD) software (Version 2.4.0.305, Thermo Fisher Scientific) and the built-in Sequest HT search engine . MS spectra lists were searched against their species-level UniProt FASTA databases (uniprot-Homo sapiens-2021-8.fasta), with Carbamidomethyl [C], TMT 6 plex(K) and TMT 6 plex (N-term) as a fixed modification and Oxi- dation (M) and Acetyl (Protein N-term) as variable modifications. The protease was trypsin. A maximum of two missed cleavage(s) was allowed. The false discovery rate (FDR) was set at 0.01 for both PSM and peptide levels. Peptide identification was performed with an initial precursor mass deviation of up to 10 ppm and a fragment mass deviation of 0.02 Da. Unique peptide and Razor peptide were used for protein quantification and total peptide amount for normalization. All the other parameters were reserved as default. The mass spectrometry proteomics data have been deposited to the ProteomeXchange Consortium via the PRIDE (Perez-Riverol et al., 2022) partner repository with the dataset identifier PXD033148.

### Bioinformatics Analysis

After pretreatment, 5,438 detected proteins were retained. Data were processed with Proteome Discoverer software (Thermo Fisher Scientific, version 2.4.0.305). Principal component analysis (PCA) was performed using the R package. (version3.6.3, https://www.r-project.org/) or SIMCA (V16.0.2, Sartorius Stedim Data Analytics AB, Umea, Sweden). Differential expressed proteins (DEPs) were defined as student’s *t*-test *p*-value < 0.05 and fold change ≤ 0.83 or fold change ≥ 1.2. DEPs were visualized in the form of volcano plot. Hierarchical clustering for representing the DEPs was conducted by R Package pheatmap. The eukaryotic clusters of orthologous groups (KOG) database (http://www.ncbi.nlm.nih.gov/COG/) of protein database was carried out for functional classification of DEPs. Gene ontology (GO) database (http://geneontology.org/) was used to classify and annotate the functions of differentially expressed proteins. All enrichment analyses were based on the Fisher’s exact test with Benjamini−Hochberg correction (*p* < 0.05).

### RNA-Seq Data Analysis

Publicly available RNA sequencing (RNA-seq) data were obtained from TCGA LUAD dataset. The clinical information and expression matrix were obtained from http://xena.ucsc.edu/ (Goldman et al., 2020). The medians of mRNA expression of PFN1 were considered as cutoffs. Survival analysis was performed by Kaplan-Meier with log-rank test. PFN1 mRNA expression in metastatic and non-metastatic LUAD patients were compared with Student *t* test.

### Statistical Analysis

All data are presented as mean ± standard deviation. Student’s *t*-test was used to compare differences between two experimental groups. For comparison of overall differences between three or more groups, one-way analysis of variance was used. Spearman correlation analysis was performed to reveal relationships between PFN1 and p-MLC. All analyses were carried out using SPSS 21.0 (IBM Corporation, Armonk, NY, United States); *p* < 0.05 was considered statistically significant.

## Results

### PFN1 Is Correlated With NSCLC Metastasis and Could Promote NSCLC Cell Migration *In Vitro*

**FIGURE 1:**
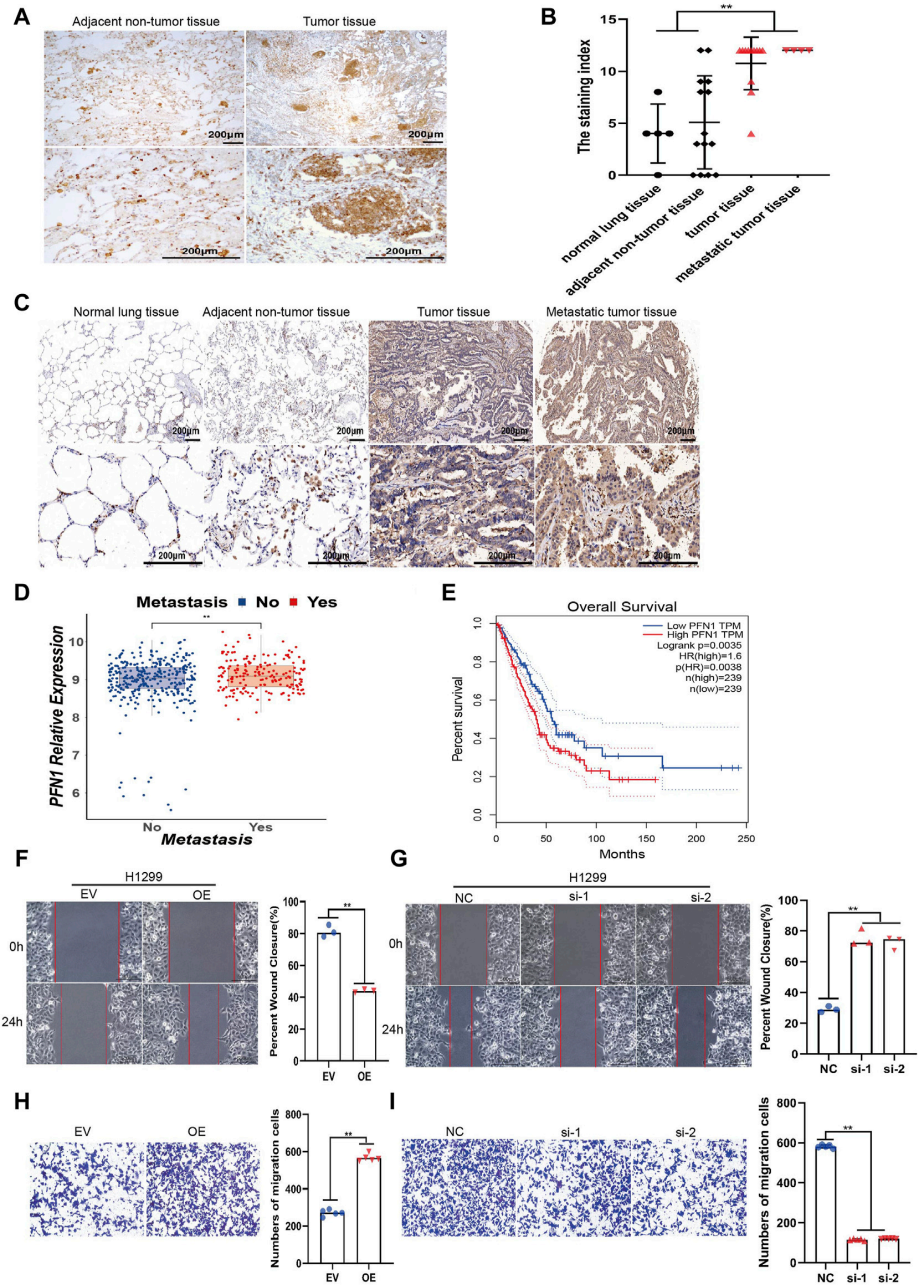
PFN1 is correlated with NSCLC metastasis and could promote NSCLC cell migration *in vitro*. **(A)** Representative IHC images of PFN1 expression on the NSCLC tissues. **(B)** The staining index of PFN1 on the tissue chip. **p < 0.01. **(C)** Representative IHC images of PFN1 expression on the tissue chip. **(D)** The expression of PFN1 in TCGA LUAD data. **p < 0.01. **(E)** The Kaplan–Meier survival analysis of PFN1 in NSCLC patients. (Data source: TCGA LUAD dataset) **(F,G)** Wound healing assays conducted to evaluate the migration ability of *PFN1*-overexpressing **(F)** and *PFN1* knockdown (KD) **(G)** H1299 cells. **p < 0.01; scale bar, 500 μm. **(H,I)** Transwell migration assays conducted to evaluate the migration of *PFN1*-overexpressing **(H)** and *PFN1* KD **(I)** H1299 cells. **p < 0.01; scale bar, 500 μm. EV, empty vector; OE, *PFN1* overexpression; NC, negative control; si-1/ 2, PFN1 siRNA1 1/2.

To investigate the roles of PFN1 in NSCLC, we first conducted IHC to detect the expression of PFN1 in NSCLC tissues. Results showed that PFN1 was highly expressed in NSCLC tissues compared with adjacent non-tumor tissues (Figure 1A). We then quantified PFN1 protein levels using tissue chip analysis. PFN1 expression was upregulated in tumor and metastatic tissues compared with that in normal lung and adjacent non-tumor tissues (Figures 1B,C; Supplementary Figure S1A), suggested that PFN1 may associated with NSCLC metastasis. Analysis of TCGA LUAD data demonstrated that PFN1 was highly expressed in patients with metastatic NSCLC (Figure 1D). The Kaplan–Meier survival analysis showed that prognosis of patients with higher expression of PFN1 was poorer than that of patients with lower expression of PFN1 (Figure 1E). These results manifested that PFN1 involves in NSCLC metastasis.

To further investigate the impact of PFN1 on NSCLC cell lines, we constructed stable PFN1-OE and PFN1-KD H1299 and A549 cell lines. The effect of overexpression and knockdown was determined using RT-qPCR (Supplementary Figures S1B,C) and western blotting (Supplementary Figure S1D). Wound healing and Transwell migration assays were conducted to determine the effect of PFN1 on migration. PFN1 overexpression promoted H1299 and A549 cell migration, whereas its downregulation inhibited cell migration (Figures 1F–I; Supplementary Figures S1E–H).

### PFN1 Could Promote MVs Secretion in NSCLC

**FIGURE 2:**
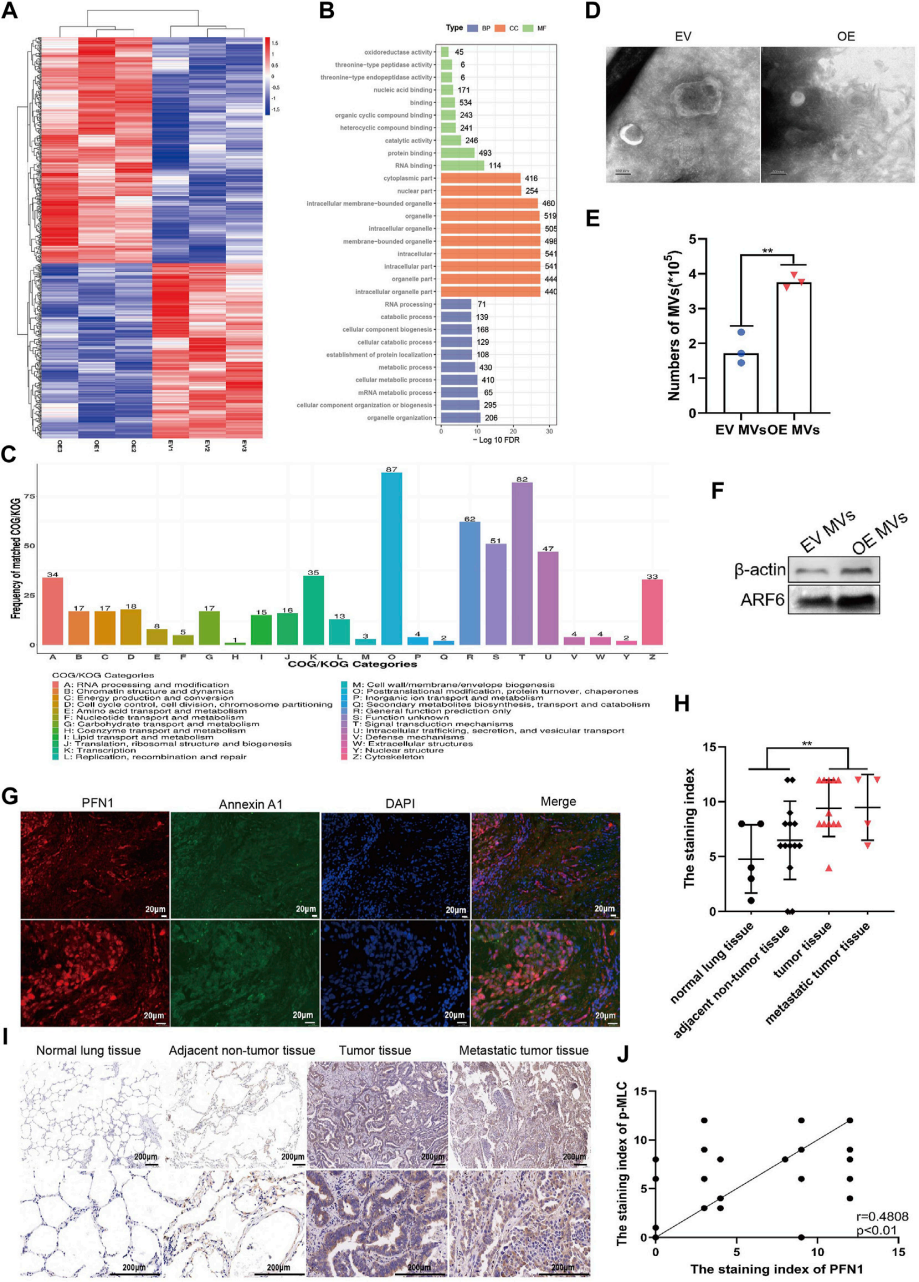
PFN1 could promote MVs secretion in NSCLC. **(A)** Heatmap of differentially expressed proteins between EV and PFN1 OE cells. **(B)** GO enrichment analysis of differentially expressed proteins. **(C)** COG/KOG analysis of differentially expressed proteins. **(D)** MVs extracted from EV-expressing and *PFN1*-overexpressing cells, using continuous differential centrifugation, identified using transmission electron microscopy. Scale bar, 100 nm. **(E,F)** Flow cytometry **(E)** and western blotting **(F)** were used to quantify MVs in *PFN1*-overexpressing and EV-expressing cells. ARF6 and actin were used as MV markers. **(G)** Expression of PFN1 and annexin A1 in lung tumor tissues detected using immunofluorescence. **(H)**The staining index of p-MLC on the tissue chip. **p < 0.01. **(I)** Representative IHC images of p-MLC expression. **(J)** Spearman rank correlation analysis was used to assess the relationship between PFN1 and p-MLC expression on the tissue chip; *p* and *r* values are shown in the plot.

To further investigate the molecular differences between EV and PFN1 OE cells and possible signaling pathways that affect NSCLC metastasis, we utilized a proteomics-based approach to characterize protein levels between them using liquid chromatography-mass spectrometry (LC-MS). Differently expressed proteins (DEPs) were defined as *p*-value < 0.05 and fold change ≤ 0.83 or fold change ≥ 1.2. We identified 581 proteins with significant differences in abundance, as shown in the volcanic plot (Supplementary Figure S2A). Compared with EV cells, 327 upregulated proteins and 254 downregulated proteins in PFN1 OE cells. The expression of DEPs was visualized with heatmap (Figure 2A). We next utilized enrichment analysis to determine if these DEPs could point to any specific biological functions that may provide insight into their differences in biological functions (Figure 2B). Among the most enriched gene ontology (GO) categories in PFN1 OE cells, DEPs involve in protein binding, cellular component organization or biogenesis and organelle organization. Most differently expressed proteins were associated with organelles, which conformed with PFN1’s roles in membrane trafficking. Cluster of orthologous groups of proteins (COG/KOG) analysis revealed that DEPs were involved in posttranslational modification, protein turnover, chaperons, intracellular trafficking, secretion, vesicular transport and signal transduction mechanisms (Figure 2C). Via proteomics analysis, we inferred that through protein interaction and signal transduction, PFN1 may participate in extracellular vesicles secretion, which in turn promote NSCLC metastasis.

As PFN1 is important in membrane trafficking and MVs are key mediators of cancer metastasis, we extracted extracellular vesicles (MVs and exosomes) from sera of clinical samples. PFN1 existed in MVs but not in exosomes or free in sera, which indicate PFN1 was correlated with MVs (Supplementary Figures S2B,C). Then we extracted MVs from EV and *PFN1*-overexpressing cell supernatants. Transmission electron microscopy, western blotting, and flow cytometry were used for qualitative and quantitative analysis of the MVs (Figures 2D–F; Supplementary Figure S2D). Results confirmed that the amount of MVs derived from PFN1 OE cells were significantly larger than that from EV cells, manifested that PFN1 could promote MVs secretion.

To further investigate the relationship between PFN1 and MVs secretion, we analyzed the expression of PFN1 and the MV marker annexin A1 (Jeppesen et al., 2019) in NSCLC tissues using immunofluorescence. PFN1 was almost co-localized with annexin A1, suggesting an association between PFN1 and annexin A1 expression and that PFN1 may participate in MV biosynthesis (Figure 2G). Phosphorylated MLC is a key molecule in the regulation of MV secretion (Muralidharan-Chari et al., 2009; Li et al., 2012; Tricarico et al., 2017). Using IHC, we determined that p-MLC levels were significantly elevated in lung cancer and metastatic tissues compared with that in adjacent tumor and normal lung tissues (Figures 2H,I; Supplementary Figure S2E). Spearman correlation analysis of PFN1 and p-MLC revealed that the expression of p-MLC was positively correlated with that of PFN1 (Figure 2J), indicating that PFN1 may regulate MLC phosphorylation, which could modulate MV secretion.

### MVs Derived From PFN1 OE Cells Promote Migration in NSCLC Cells

**FIGURE 3:**
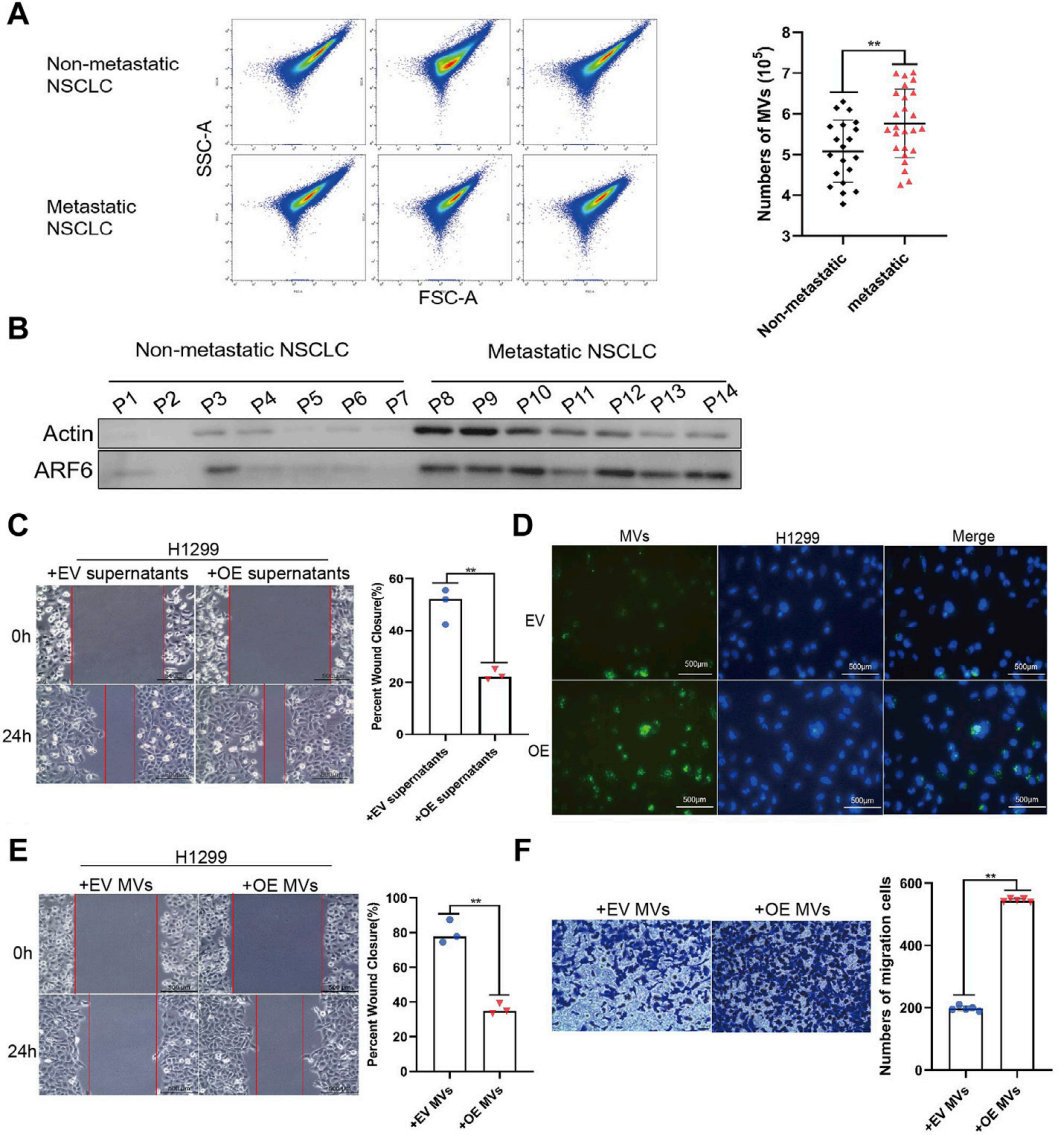
MVs derived from PFN1 OE cells promote migration in NSCLC cells. **(A)** MVs collected from sera of patients with NSCLC quantified using flow cytometry. **p < 0.01. **(B)** Protein expression of ARF6 and β-actin in MVs collected from sera of patients with NSCLC detected using western blotting. **(C)** Effect of *PFN1*-overexpressing cell supernatants on cell migration evaluated through wound healing assays. **p < 0.01; scale bar, 500 μm. **(D)** PKH67-labeled MVs taken up by H1299 cells. DAPI was used to stain the nuclei of H1299 cells. Scale bar, 500 μm. **(E,F)** Wound healing **(E)** and Transwell migration **(F)** assays conducted to evaluate the migration of H1299 cells after treatment with MVs derived from EV-expressing and *PFN1*-overexpressing cells; **p < 0.01; scale bar, 500 μm.

MVs are key mediators of cancer metastasis. We collected serum samples from patients with metastatic (*n* = 25) and non-metastatic (*n* = 20) lung cancer and extracted MVs. Flow cytometry (100–1,000 nm microparticles analyzed) and western blotting (ARF6 used as MV biomarker) (Jeppesen et al., 2019) results indicated that the number of MVs in patients with metastatic NSCLC was higher than that in patients with non-metastatic NSCLC (Figures 3A,B). To determine whether PFN1 promotes NSCLC migration by inducing the secretion of mediators into the environment, we treated H1299 and A549 cells with supernatants of cultured EV-expressing or *PFN1*-overexpressing H1299 and A549 cells and performed a wound healing assay. The *PFN1*-overexpressing cell supernatants significantly promoted cell migration, which was unaffected by EV-expressing cell supernatants (Figure 3C and Supplementary Figure S3A). To visualize the uptake of MVs by cells, we used PKH67 to label MVs derived from EV-expressing and *PFN1*-overexpressing cells. Immunofluorescence showed that MVs derived from *PFN1*-overexpressing cells were more abundant than those derived from EV-expressing cells. In addition, MVs were taken up by H1299 cells (Figure 3D). We then treated H1299 and A549 cells with EV-expressing and *PFN1*-overexpressing cell-derived MVs and found that the latter significantly promoted H1299 and A549 cell metastasis, according to wound healing and Transwell migration assays (Figures 3E,F; Supplementary Figures S3B,C). Hence, PFN1 may promote NSCLC metastasis through the induction of MV secretion.

### PFN1 Promotes *In Vivo* NSCLC Metastasis by Elevating MV Secretion

**FIGURE 4:**
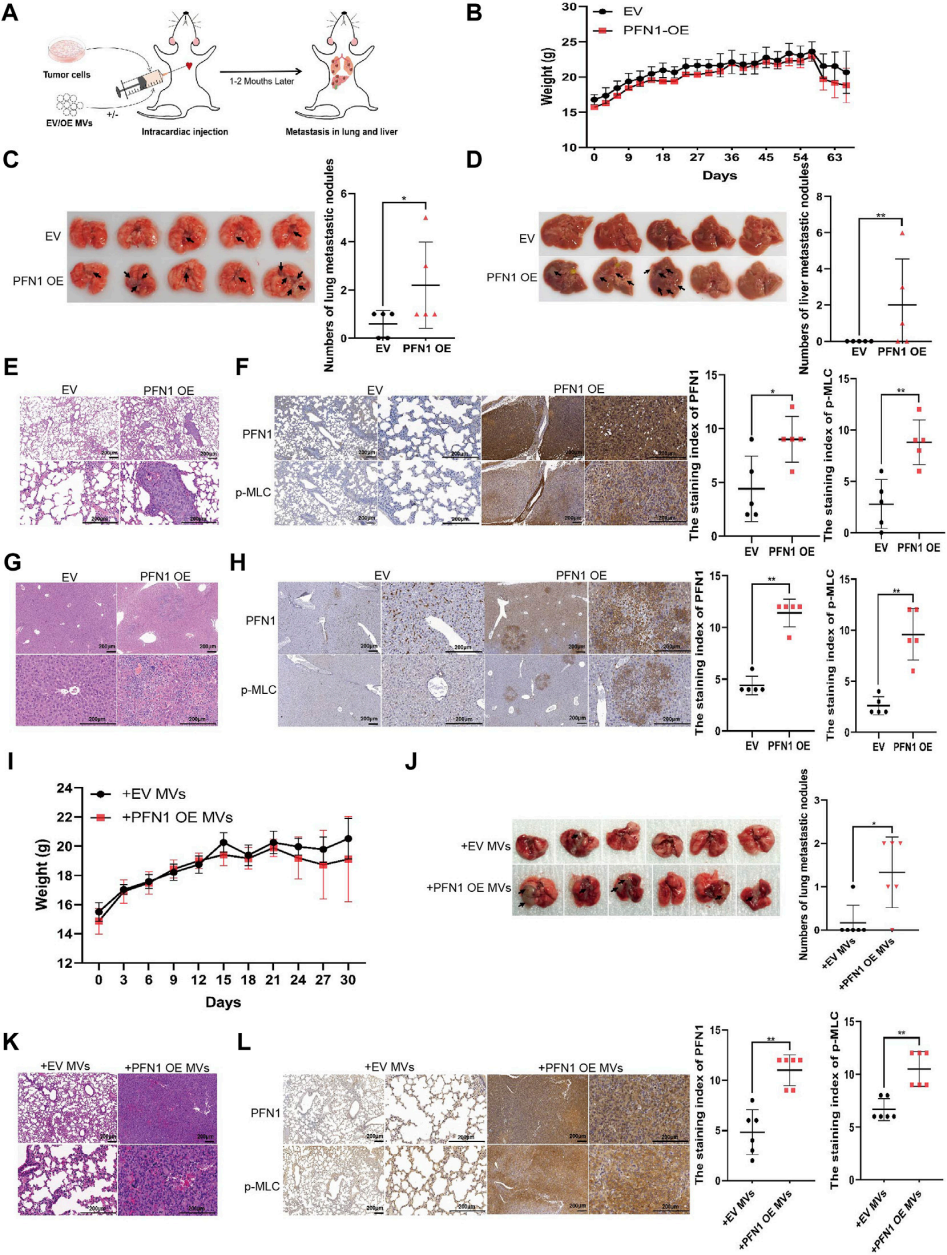
PFN1 promotes *in vivo* NSCLC metastasis by elevating MV secretion. **(A)** Schematic illustration of the mouse model of metastatic tumor established to determine the role of PFN1 in tumor metastasis. **(B)** Body weight changes in mice after intracardiac injection of *PFN1*-overexpressing and EV-expressing cell lines. **(C,D)** Representative images of lung **(C)** and liver **(D)** metastases of the mouse model. The number of metastases is displayed in the right-hand side graph. *p < 0.05, **p < 0.01. **(E)** Representative images of HE-stained lung tissues of the mouse model. **(F)** Representative IHC images of PFN1 and p-MLC expression in lung tissues. The staining index is shown in the right-hand side graph. **p < 0.01. **(G)** Representative images of HE-stained liver tissues of the mouse model. **(H)** Representative IHC images of PFN1 and p-MLC expression in liver tissues. The staining index is shown in the right-hand side graph. **p < 0.01. **(I)** Body weight changes in mice after intracardiac injection of H1299 cells and MVs. **(J)** Representative images of lung metastases of the mouse model. The number of metastases is shown in the bottom graph. *p < 0.05. **(K)** Representative images of HE-stained lung tissues of the mouse model. **(L)** Representative IHC images of PFN1 and p-MLC expression in lung tissues. The staining index is shown in the right-hand side graph; *p < 0.05.

To further investigate the role of PFN1 in NSCLC metastasis, we established a mouse model of tumor metastasis via intracardiac injection of H1299 NSCLC cells (Figure 4A). The body weights of mice are shown in Figure 4B. PFN1 overexpression promoted lung (Figure 4C) and liver (Figure 4D) metastasis in mice. Representative images of hematoxylin and eosin (HE)-stained lung tissues are shown in Figure 4E. IHC showed that PFN1 and p-MLC expression in the lung tissue of *PFN1*-overexpressing cell-treated group was higher than in the EV-expressing cell-treated group (Figure 4F). Representative images of hematoxylin and eosin (HE)-stained liver tissues are shown in Figure 4G. IHC also showed that PFN1 and p-MLC expression in the liver tissues of *PFN1*-overexpressing cell-treated group was higher than in the EV-expressing cell-treated group (Figure 4H).

The effects of *PFN1*-overexpressing cell-derived MVs on NSCLC metastasis were also similar to those observed in the *in vitro* assays. The body weight of mice is shown in Figure 4I. Compared with EV-expressing cell-derived MVs, *PFN1*-overexpressing cell-derived MVs promoted H1299 cell metastasis to the lungs (Figure 4J). HE results of lung tissues support these findings (Figure 4K). IHC analysis revealed that PFN1 and p-MLC expression in the lung tissues of the group treated with *PFN1*-overexpressing cell-derived MVs was higher than that in the group treated with EV-expressing cell-derived MVs (Figure 4L). These results further confirmed the role of PFN1 in NSCLC metastasis.

### Mechanisms Underlying the Promotion of MLC Phosphorylation by PFN1

**FIGURE 5:**
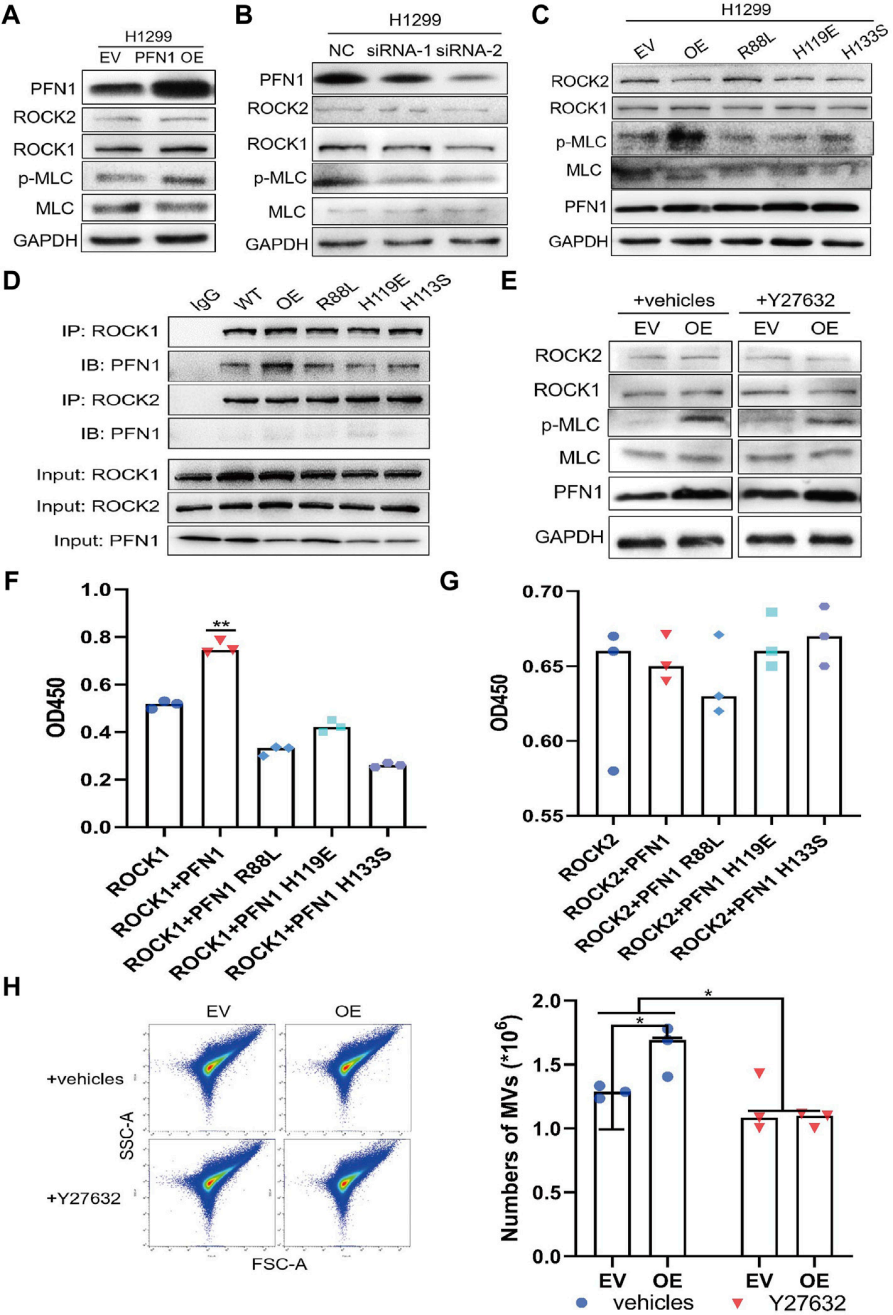
Mechanisms underlying the promotion of MLC phosphorylation by PFN1. **(A,B)** Protein expression after *PFN1* overexpression **(A)** and knockdown **(B)** measured using western blotting. **(C)** Protein expression in PFN1 mutants measured using western blotting. **(D)** PFN1 interactions with ROCK1/2 confirmed using co-IP. **(E)** Protein expression after treatment with Y27632 (10 µM) measured using western blotting. **(F)** Effect of PFN1 on ROCK1 activity. **p < 0.01. **(G)** Effect of PFN1 on ROCK2 activity. **(H)** Flow cytometry measuring changes in the amount of MVs after treatment with Y27632; *p < 0.05.

Previous studies have shown that the ARF6/ERK/p-MLC and ROCK/p-MLC pathways are involved in MV secretion (Li et al., 2012; Muralidharan-Chari et al., 2009). ARF6 and ERK (extracellular signal-regulated kinase 1/2) expression were not affected by PFN1 expression (Supplementary Figure S4A). As ROCK1/2 play important roles in actin cytoskeleton organization (Goldman et al., 2020), we speculated that PFN1 interacts with ROCK1/2 to indirectly regulate MV secretion. We, therefore, analyzed ROCK1/2, MLC, and p-MLC expression in *PFN1*-overexpressing and knockdown cells. PFN1 overexpression promoted MLC phosphorylation, whereas PFN1 knockdown inhibited it (Figures 5A,B and Supplementary Figures S4B,C). The protein expression of ROCK1/2 remained unchanged when PFN1 overexpression or downregulation. When we assessed MLC phosphorylation in cell lines expressing the PFN1 mutants R88L, H119E, or H133S (mutated PIP2/PIP3-binding site, actin-binding site, and PLP-binding site, respectively) (Sohn et al., 1995; Suetsugu et al., 1998; Julian and Olson, 2014), and observed a decrease in MLC phosphorylation with all three mutants (Figure 5C and Supplementary Figure S4D).

We next conducted a co-IP assay to characterize the interactions among ROCK1/2, PFN1, and its mutants. ROCK1 interacted with wildtype, not mutant PFN1, while ROCK2 did not interact with PFN1 (Figure 5D). Y27632 is an inhibitor of ROCK activity and MV secretion (Björkegren-Sjögren et al., 1997; Catalano et al., 2020). Western blotting results showed that treatment with Y27632 reduced MLC phosphorylation in *PFN1*-overexpressing cells but did not affect ROCK1/2 expression (Figure 5E and Supplementary Figure S4E).

We also overexpressed ROCK1 and ROCK2 in HEK-293T cells and purified ROCK1/2, PFN1, and its mutant proteins to conduct a ROCK kinase assay *in vitro* (Supplementary Figures S4F,G). Wildtype PFN1 enhanced ROCK1 activity, whereas PFN1 mutants reduced it (Figure 5F). PFN1 exerted no effect on ROCK2 activity (Figure 5G). Immunofluorescence confirmed the co-location of PFN1 and ROCK1 in H1299 cells. (Supplementary Figures S4H,I) Flow cytometry showed that after treatment with Y27632 (10 µM), the amount of MVs decreased in PFN1-overexpressing cells (Figure 5H). Hence, PFN1 can interact with ROCK1, enhance its kinase activity, and indirectly promote MLC phosphorylation to ultimately induce MV secretion. Y27632 partly reversed the effect of PFN1 in promoting MLC phosphorylation and MV secretion.

### ROCK1 Inhibitor Y27632 Partially Reverses the Effect of PFN1 on NSCLC Metastasis *In Vitro* and *In Vivo*

**FIGURE 6:**
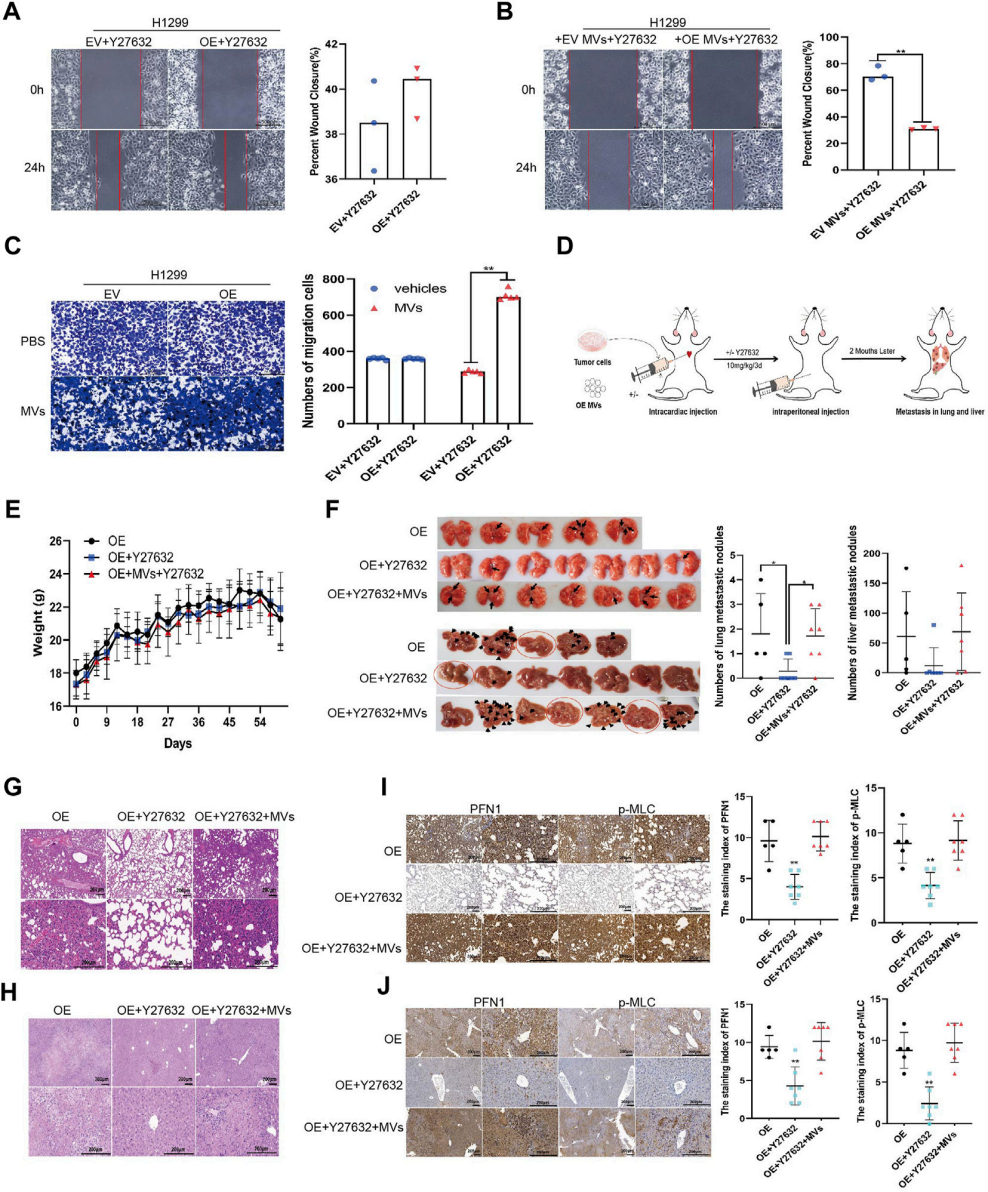
ROCK1 inhibitor Y27632 partially reversed the promotion of lung cancer metastasis by PFN1 *in vitro* and *in vivo*. **(A,B)** Wound healing assays conducted to evaluate the effect of Y27632 **(A)** and Y27632 combined with MVs **(B)** on cell migration. **p < 0.01; scale bar, 500 μm. **(C)** Transwell migration assays conducted to evaluate the effect of Y27632 and Y27632 combined with MVs on cell migration. **p < 0.01; scale bar, 500 μm. **(D)** Schematic diagram of the mouse model of metastatic tumor established to determine the effect of Y27632 on PFN1-induced lung cancer metastasis. **(E)** Body weight changes in mice after intracardiac injection of *PFN1*-overexpressing H1299 cells and intraperitoneal injection of Y27632 (10 mg/kg). **(F)** Representative images of lung and liver metastatic tissue in mice. The number of metastatic nodules is shown in the right-hand side graph. *p < 0.05. **(G,H)** Representative images of HE-stained lung **(G)** and liver **(H)** metastases. **(I)** Representative IHC images of PFN1 and p-MLC expression in lung tissues. The staining index is shown in the right-hand side graph. **p < 0.01. **(J)** Representative IHC images of PFN1 and p-MLC expression in liver tissues. The staining index is shown in the right-hand side graph; **p < 0.01.

The wound healing assay showed that migration of Y27632-treated, *PFN1*-overexpressing cells diminished to nearly that of the EV-expressing cells (Figure 6A and Supplementary Figure S5A). Next, we added *PFN1*-overexpressing cell-derived MVs to Y27632-treated cells and found that Y27632 did not reverse the migration induced by *PFN1*-overexpressing cell-derived MVs (Figure 6B and Supplementary Figure S5B). Similar results were obtained from the Transwell migration assay (Figure 6C and Supplementary Figure S5C).

We also validated these results using our mouse model (Figure 6D; body weight: Figure 6E). After treatment with Y27632 (10 mg/kg every 3 days, intraperitoneal injection), the number of metastatic nodules in the lungs and livers of mice significantly decreased compared with mice injected with *PFN1*-overexpressing cells alone. However, when mice were simultaneously injected with *PFN1*-overexpressing cell-derived MVs, the number of metastatic nodules in the lung and liver markedly increased compared with the group treated with Y27632 (Figure 6F). HE-staining displaying the micromorphology of tumor nodules are presented in Figures 6G,H. IHC analysis revealed higher PFN1 and p-MLC expression in both lung and liver tissues of the group treated with *PFN1*-overexpressing cells and *PFN1*-overexpressing cells + MVs + Y27632 compared to those treated with Y27632 alone (Figures 6I,J).

**FIGURE 7:**
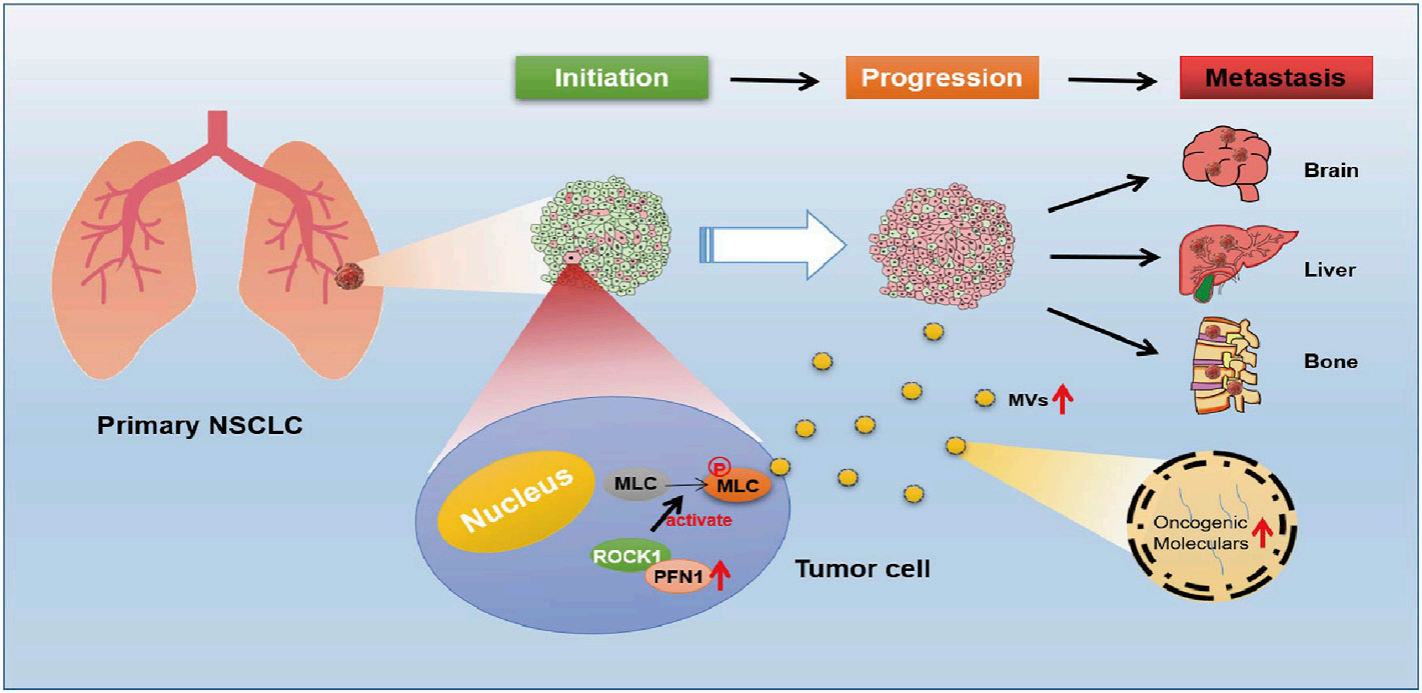
Schematic diagram of the role of PFN1 in NSCLC metastasis. In the initiation stage of NSCLC, cells with upregulated PFN1 secret more MVs through PFN1 interactions with the ROCK/p-MLC pathway. These MVs contain numerous oncogenenic moleculars, which could enhance migration abilities of PFN1 normal expressed NSCLC cells, and untimately promote progression and metastasis of NSCLC.

A schematic illustration of the role played by PFN1 in NSCLC metastasis is shown in Figure 7. During metastasis initiation, *PFN1*-overexpressing NSCLC cells secrete more MVs through PFN1 interactions with the ROCK/p-MLC pathway. These MVs deliver tumorigenic bioactive substances to recipient cells and enhance their migratory ability, thus promoting NSCLC progression.

## Discussion

Intratumor heterogeneity is a key contributor for therapeutic failure and drug resistance of cancer patients. (Greaves, 2015).Early detection of abnormal expression of genes and proteins thus critical for precision medicine. (Smida and Nijman, 2012; McGranahan and Swanton, 2017).In this study, we explored the role of PFN1 in NSCLC metastasis and found that PFN1 is highly expressed in advanced NSCLC tissues. Upregulation of PFN1 was correlated with worse prognosis of patients with NSCLC. PFN1 expression of patients with metatatic NSCLC was significantly higher than that of patients with non-metastatic NSCLC. These results suggested that detection of PFN1 expression in NSCLC tissues may help predict prognosis and guide early intervention of NSCLC. *In vitro* experiments confirmed that PFN1 overexpression promoted NSCLC cell migration, while its downregulation inhibited NSCLC cell migration. Previous studies pertaining to the role of PFN1 in cancer metastasis have yielded contrasting results, PFN1 exhibits variable effects on the metastasis of different tumors. For example, PFN1 promotes the metastasis of breast cancer (Ding et al., 2014) and hepatocellular carcinoma (Wang et al., 2019), while PFN1 inhibits the metastasis of bladder cancer (Frantzi et al., 2016). The mechanisms underlying the roles of PFN1 in metastasis were different from cancer to cancer and those in lung cancer are still unclear. In this study, we found that PFN1 could significantly promote MV secretion in NSCLC cells, which instigated us to study its roles in NSCLC metastasis.

Tumor-cell derived MVs play important roles in tumor development and progression (Muralidharan-Chari et al., 2010). Proteomics analysis reveals PFN1 was closely associated with membrane trafficking. Further COG/KOG analysis inferred that PFN1 involved in intracellular trafficking and vesicles secretion. *In vitro* experiments showed that PFN1 was positively correlated with MLC phosphorylation, a key process in the shedding of MVs (Tricarico et al., 2017). PFN1 overexpression significantly promoted MLC phosphorylation and increased MV secretion from NSCLC cells. MVs derived from PFN1 OE cells significantly promote NSCLC metastasis *in vitro* and *in vivo*. Dysregulation of PFN1 in cells affect surrounding cells, even distant cells, via cell communication mediated by MVs. Through regulation of MVs secretion, PFN1 exerts its positive roles in NSCLC metastasis.

In this study, PFN1 could interact with ROCK1 followed by its activation, thus promoting MLC phosphorylation. Interestingly, only wild type PFN1 could interact with ROCK1. All three mutants of PFN1 displayed no interaction with ROCK1 and even exerted inhibitory effect on ROCK1 activities. ROCK1 activity can be regulated through interaction with common activators (such as the small Rho GTPases) (Wei et al., 2016) and plasma membrane via its C-terminal PH domain (Fu et al., 1998; Feng et al., 1999). Changes in the plasma membrane structures induced by PFN1 explain ROCK1 activation by PFN1 (Davey and Moens, 2020). Mutations in PFN1 binding domains lead to radical 3D structure changes of PFN1, which could ultimately affect the interaction of PFN1 with other proteins (Boopathy et al., 2015). This may explain why the interactions between PFN1 and ROCK1 require an intact PFN1. Besides, abnormal activation of ROCK1 can enhance tumor cell migration (Wei et al., 2016). Moreover, PFN1 is a direct target of ROCK1 (Shao et al., 2008). Epigenetic regulation of PFN1 could also affect functions of PFN1, thus regulated interation between PFN1 and ROCK1 (Brahma et al., 2010). The interactions between PFN1 and ROCK1 may be much more complex *in vivo* and play important roles in cancer progression, which needs further investigation in the future.

Signaling pathways regulated by the small Ras-related GTPases (ARF6 and Rho family) are thought to govern the formation and release of tumor-derived MVs. All these molecules ultimately regulate the shedding of MVs through the phosphorylation and activation of MLC (Muralidharan-Chari et al., 2009; Li et al., 2012). The elongation and organization of actin filament along the plasma membrane are essential for MV formation, in which several cytoskeletal proteins, such as cofilin (Li et al., 2012) and formin (Di Vizio et al., 2009), are involved. In this study, we highlight the role of PFN1 in the RhoA/ROCK signaling pathway for the formation of MVs. PFN1 may participate in the formation of MVs by regulating the polymerization and depolymerization of actin filament. In addition to the regulation of actin cytoskeleton, PFN1 may be involved in MV biogenesis and secretion, which warrants further investigations.

In conclusion, our findings suggest that PFN1, a critical actin-regulating protein, promotes MV release through the ROCK/p-MLC pathway, thereby promoting NSCLC metastasis. Thus, PFN1 may represent a potential therapeutic target for NSCLC metastasis. By reducing the release of MVs, it may be possible to partially reverse PFN1 overexpression-induced NSCLC cell migration. This study provides a potential new approach for the treatment of NSCLC by targeting metastasis that warrants further investigation.

## Data Availability

The datasets presented in this study can be found in online repositories. The name of the repository and accession number can be found below: ProteomeXchange; PXD033148.
