# Supplementary material for: Profilin 1 Induces Tumor Metastasis by Promoting Microvesicle Secretion Through the ROCK 1/p-MLC Pathway in Non-Small Cell Lung Cancer
Source: Front Pharmacol. 2022 May 2;13:890891. doi: 10.3389/fphar.2022.890891 (PMC9108340; doi:10.3389/fphar.2022.890891)
Supplement: Supplementary file 1 [file Table1.docx]

**Supplementary Tables**

**Table S1. Patient information.**

| **Number** | **Patients' ID** | **Sex** | **Age** | **Tumor location** | **Tumor**  **size (cm)** | **Pathological**  **type** | **Distant metastasis** | **TNM stage** | **Clinical**  **stage** |
| --- | --- | --- | --- | --- | --- | --- | --- | --- | --- |
| 1 | 0004610272 | M^a^ | 74 | left upper lobe | 6*4*3.5 | LUAD^b^ | - | pT3N0M0 | IIB |
| 2 | 0005925652 | M^c^ | 55 | right upper lobe | 1.5 | LUAD | - | pT1bN0M0 | IA2 |
| 3 | 0008539361 | F | 60 | left upper lobe | 2*1.5 | LUAD | - | T2aN0M0 | IB |
| 4 | 0008272307 | F | 57 | right lower lobe | 0.8 | LUAD | - | sT1N0M0 | IA1 |
| 5 | 0001754946 | M | 47 | right upper lobe | 2*2*0.8 | LUAD | - | pT1bN0M0 | IA2 |
| 6 | 0006484007 | M | 45 | right lower lobe | 0.8 | LUAD | - | pT1miN0M0 | IA1 |
| 7 | 0008574022 | F | 55 | right upper lobe | 0.8*0.9 | LUAD | - | pTisN0M0 | IA1 |
| 8 | 0007887771 | F | 50 | right lower lobe | 1.5*2 | LUAD | - | pT1bN0M0 | IA2 |
| 9 | 0005615881 | F | 59 | right lower lobe | 2.6*2.6 | LUAD | - | pT1cN0M0 | IA3 |
| 10 | 0005485241 | F | 51 | right lower lobe | 3*3 | LUAD | - | pT1cN0M0 | IA3 |
| 11 | 0003079868 | F | 56 | right upper lobe | 1.5*2 | LUAD | - | pT2(visPI)N0Mx | IB |
| 12 | 0006206276 | F | 67 | left lower lobe | 1.3*1.1 | LUAD | - | carcinoma in situ | IA2 |
| 13 | 0008609036 | F | 65 | right middle lobe | 1.5 | LUAD | - | pT1bN0Mx | IA2 |
| 14 | 0008481860 | F | 63 | right upper lobe | 4 | LUAD | - | pT1cN1aMx | IA3 |
| 15 | 0004400805 | F | 72 | left lower lobe | 2.5 | LUAD | - | pT1cN0M0 | IA3 |
| 16 | 0008657133 | F | 54 | right upper lobe | 0.6*0.8; 0.3*0.4 | LUAD | - | T1a(mi)N0Mx | IA1 |
| 17 | 0008649096 | F | 51 | right upper lobe | 1.5*1.2 | LUAD | - | pT1bN0Mx | IA2 |
| 18 | 0005925652 | M | 65 | right upper lobe | 1.5 | LUAD | - | pT1bN0M0 | IA2 |
| 19 | 0008647552 | M | 63 | right upper lobe | 3.6*2.5 | LUAD | - | pT1cN0Mx | IA3 |
| 20 | 0008478178 | F | 60 | right lower lobe | 2.5*2 | LUAD | - | pT1cN0M0 | IA3 |
| 21 | 0008542650 | M | 54 | right lung | 4.0*6.1(CT) | LUAD | left adrenal gland, bones, brain | T4N3M1c | IVB |
| 22 | 0001530820 | F | 41 | left lung | 1.6*1(CT) | LUAD | pleura, lymph nodes, bones | T4N3M1c | IVB |
| 23 | 0008566553 | M | 72 | left upper lobe |  | LUAD | lymph nodes | T3N3Mx | IIIC |
| 24 | 0008502076 | M | 73 | left lower lobe | 5.2*3.4(CT) | LUAD | lymph nodes, bones | T3N3M1b | IVA |
| 25 | 0008514106 | M | 61 | right lower lobe | 4.1*4.1(CT) | LUAD | brain, adrenal gland | T4N2M1c | IVB |
| 26 | 0008497950 | F | 53 | right lower lobe | 1.6*1.8(CT) | LUAD | lymph nodes, bones, brain | T1cN3M1c | IVB |
| 27 | 0002266910 | M | 55 | left upper lobe |  | LUAD | lungs | T2centerN2M1a | IVA |
| 28 | 0002843488 | M | 71 | left upper lobe | 0.54*0.44(CT) | LUAD | bones, liver, lymph nodes | T4N3M1c | IVB |
| 29 | 0008542650 | M | 54 | right upper lobe | 4.0*6.1 | LUAD | adrenal gland, bones, brain, lymph nodes | T4N3M1c | IVB |
| 30 | 0008580789 | F | 72 | right upper lobe |  | LUAD | liver, bones, pleura, lymph nodes | T4N3M1c | IVB |
| 31 | 0008565532 | F | 54 | right lung |  | LUAD | lung, bones, lymph nodes, brain | T4N3M1c | IVB |
| 32 | 0007897841 | M | 49 | left lower lobe |  | LUAD | bones | T2N0M1c | IVB |
| 33 | 0008350078 | F | 50 | right lower lobe |  | LUAD | lungs, lymph nodes, bones | T4N1M1b | IVA |
| 34 | 0008610790 | M | 31 | right middle lobe | 0.31*0.21(CT) | LUAD | brain, pleura | T4N1M1b | IVA |
| 35 | 00085546274 | M | 52 | right lower lobe |  | LUAD | diaphragm，pericardium | TXNXM1 | IV |
| 36 | 0005818574 | M | 54 | left lower lobe and right upper lobe |  | LUAD | bones | T4N0M1b | IVA |
| 37 | 0008134908 | M | 57 | left lower lobe |  | LUAD | lungs, bones, lymph nodes | T4N3M1b | IVA |
| 38 | 0008602981 | F | 56 | left upper lobe | 2.9*3.5;2.9*2.7(CT) | LUAD | lymph nodes, pleural, bones | T4N3M1c | IVB |
| 39 | 0008103464 | M | 49 | left lower lobe |  | LUAD | lungs | T4N0M1a | IVA |
| 40 | 0008639558 | F | 57 | left upper lobe |  | LUAD | lymph nodes, bones | T1cN3M1c | IVB |
| 41 | 0008681152 | M | 73 | left upper lobe | 3.6*2.6(CT) | LUAD | lung, bones, lymph nodes, pleural | T2aN3M1c | IVB |
| 42 | 0008646448 | M | 64 | right lung | 0.18(CT) | LUAD | pleural | TxN0M1a | IVA |
| 43 | 0008410270 | M | 62 | right upper lobe |  | LUAD | lymph nodes, adrenal gland, bones | T2N3M1c | IVB |
| 44 | 0006454091 | M | 64 | right lung |  | LUAD | pleural, bones, brain, lymph nodes | T2N2M1c | IVB |
| 45 | 0008584475 | M | 70 | left lower lobe |  | LUAD | lymph nodes, pleural, bones, liver, peritoneum | T2N3M1c | IVB |

Footnotes: a, M: male, b, LUAD: lung adenocarcinoma, c, F: female

**Table S2. Detailed information about the tissue chip.**


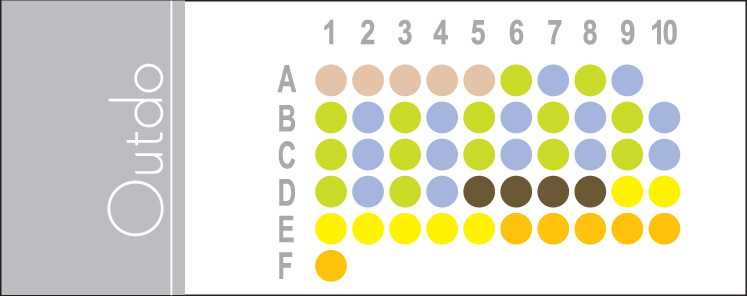


| **Spot** | **Codes of paraffin mass** | **Tissue sources** | **Pathological type** | **TNM stage** | **Clinical stage** |
| --- | --- | --- | --- | --- | --- |
| A1 | N0001E0500-B31-1 | healthy control lung | healthy control lung tissue |  |  |
| A2 | N0002E0500-B31-1 | healthy control lung | healthy control lung tissue |  |  |
| A3 | N0004E0500-B31-1 | healthy control lung | healthy control lung tissue |  |  |
| A4 | N0007E0500-B31-1 | healthy control lung | healthy control lung tissue |  |  |
| A5 | N0008E0500-B31-1 | healthy control lung | healthy control lung tissue |  |  |
| A6 | E05A0330-B31-P1 | adjacent tumor tissue | lung tissue | T1bN0M0 | 1A |
| A7 | E05A0330-B31-C1 | Primary LUAD | LUAD with squamous metaplasia |  |  |
| A8 | E05A0150-B31-P1 | adjacent tumor tissue | lung tissue | T2aN0M0 | 1B |
| A9 | E05A0150-B31-C1 | Primary LUAD | LUAD |  |  |
| A10 |  | | | | |
| B1 | E05A0248-B31-P1 | adjacent tumor tissue | lung tissue | T2aN0M0 | 1B |
| B2 | E05A0248-B31-C1 | Primary LUAD | LUAD |  |  |
| B3 | E05A0739-B31-P1 | adjacent tumor tissue | lung tissue | T1bN1M0 | 2A |
| B4 | E05A0739-B31-C1 | Primary LUAD | LUAD |  |  |
| B5 | E05A0772-B31-P1 | adjacent tumor tissue | lung tissue | T2aN1M0 | 2A |
| B6 | E05A0772-B31-C1 | Primary LUAD | LUAD |  |  |
| B7 | E05A0334-B31-P1 | adjacent tumor tissue | lung tissue | T2aN2M0 | 3A |
| B8 | E05A0334-B31-C1 | Primary LUAD | LUAD |  |  |
| B9 | E05A0024-B31-P1 | adjacent tumor tissue | lung tissue | T2aN2M0 | 3A |
| B10 | E05A0024-B31-C1 | Primary LUAD | LUAD |  |  |
| C1 | E05A0120-B31-P1 | adjacent tumor tissue | lung tissue | T2aN2M0 | 3A |
| C2 | E05A0120-B31-C1 | Primary LUAD | LUAD |  |  |
| C3 | E05A0205-B31-P1 | adjacent tumor tissue | lung tissue | T2aN3M0 | 3B |
| C4 | E05A0205-B31-C1 | Primary LUAD | LUAD |  |  |
| C5 | E05A0291-B31-P1 | adjacent tumor tissue | lung tissue | T4NxM0 | 3 |
| C6 | E05A0291-B31-C1 | Primary LUAD | LUAD with squamous metaplasia |  |  |
| C7 | E05A2163-B31-P1 | adjacent tumor tissue | lung tissue | TxNxM1b | 4 |
| C8 | E05A2163-B31-C1 | Primary LUAD | LUAD |  |  |
| C9 | E05A0196-B31-P1 | adjacent tumor tissue | lung tissue | T2aN2M1b | 4 |
| C10 | E05A0196-B31-C1 | Primary LUAD | LUAD |  |  |
| D1 | E05A0952-B31-P1 | adjacent tumor tissue | lung tissue | T3N2M1b | 4 |
| D2 | E05A0952-B31-C1 | Primary LUAD | LUAD |  |  |
| D3 | E05A0997-B31-P1 | adjacent tumor tissue | lung tissue | T2aN0M1b | 4 |
| D4 | E05A0997-B31-C1 | Primary LUAD | LUAD |  |  |
| D5 | P01A0132-B31-C1 | Metastasis of LUAD | Metastatic LUAD |  |  |
| D6 | P01A0135-B31-C1 | Metastasis of LUAD | Metastatic LUAD |  |  |
| D7 | P01A0099-B31-C1 | Metastasis of LUAD | Metastatic LUAD |  |  |
| D8 | E06A0018-B31-C1 | Metastasis of LUAD | Metastatic LUAD |  |  |
| D9 | E05A0243-B31-L1 | lymph nodes of LUAD | negative lymph node |  |  |
| D10 | E05A0325-B31-L1 | lymph nodes of LUAD | negative lymph node |  |  |
| E1 | E05A0326-B31-L1 | lymph nodes of LUAD | negative lymph node |  |  |
| E2 | E05A0378-B31-L1 | lymph nodes of LUAD | negative lymph node |  |  |
| E3 | E05A0379-B31-L1 | lymph nodes of LUAD | negative lymph node |  |  |
| E4 | E05A0397-B31-L1 | lymph nodes of LUAD | negative lymph node |  |  |
| E5 | E05A0599-B31-L1 | lymph nodes of LUAD | negative lymph node |  |  |
| E6 | E05A0174-B31-L1 | lymph nodes of LUAD | positive lymph node |  |  |
| E7 | E05A0260-B31-L1 | lymph nodes of LUAD | positive lymph node |  |  |
| E8 | E05A0324-B31-L1 | lymph nodes of LUAD | positive lymph node |  |  |
| E9 | E05A0507-B31-L1 | lymph nodes of LUAD | positive lymph node |  |  |
| E10 | E05A0731-B31-L1 | lymph nodes of LUAD | positive lymph node |  |  |
| F1 | E05A0783-B31-L1 | lymph nodes of LUAD | positive lymph node |  |  |

| **tissue type** | **code of tissue** | **organ** | **sex** | **Age**  **(year)** | **distant metastasis** | **phathogy type** | **clinical stage** | **tumor size(cm)** | **tumor location** |
| --- | --- | --- | --- | --- | --- | --- | --- | --- | --- |
| healthy control lung | N0001E0500 | lung | male | Adult | —— | healthy control lung | —— | —— | —— |
| healthy control lung | N0002E0500 | lung | male | Adult | —— | healthy control lung | —— | —— | —— |
| healthy control lung | N0004E0500 | lung | male | Adult | —— | healthy control lung | —— | —— | —— |
| healthy control lung | N0007E0500 | lung | male | Adult | —— | healthy control lung | —— | —— | —— |
| healthy control lung | N0008E0500 | lung | male | Adult | —— | healthy control lung | —— | —— | —— |
| adjacent tumor tissue/Primary lung adenocarcinoma | E05A0330 | lung | female | 68 | non | lung adenocarcinoma with squamous metaplasia | **Ⅱ** | 3×3×1.5cm | left lower lobe |
| adjacent tumor tissue/Primary lung adenocarcinoma | E05A0150 | lung | female | 63 | non | lung adenocarcinoma | **Ⅱ-Ⅲ** | 4×3.5×2cm | left upper lobe |
| adjacent tumor tissue/Primary lung adenocarcinoma | E05A0248 | lung | male | 60 | non | lung adenocarcinoma | **Ⅲ** | 4×3×2cm | left upper lobe |
| adjacent tumor tissue/Primary lung adenocarcinoma | E05A0739 | lung | male | 60 | non | lung adenocarcinoma | **Ⅱ-Ⅲ** | 2.5×2×2.5cm | left upper lobe |
| adjacent tumor tissue/Primary lung adenocarcinoma | E05A0772 | lung | female | 60 | non | lung adenocarcinoma | **Ⅲ** | 3.5×3×2.5cm | left lower lobe |
| adjacent tumor tissue/Primary lung adenocarcinoma | E05A0334 | lung | female | 75 | non | lung adenocarcinoma | **Ⅱ** | 2.5×1.5×1.3cm | right lower lobe |
| adjacent tumor tissue/Primary lung adenocarcinoma | E05A0024 | lung | male | 52 | non | lung adenocarcinoma | **Ⅱ-Ⅲ** | 3×3×3cm | left upper lobe |
| adjacent tumor tissue/Primary lung adenocarcinoma | E05A0120 | lung | female | 50 | non | lung adenocarcinoma | **Ⅱ-Ⅲ** | 3.5×2.5×1.5cm | left upper lobe |
| adjacent tumor tissue/Primary lung adenocarcinoma | E05A0205 | lung | male | 56 | non | lung adenocarcinoma | **Ⅱ** | 3×2.5×2.5cm | right upper lobe |
| adjacent tumor tissue/Primary lung adenocarcinoma | E05A0291 | lung | male | 45 | non | lung adenocarcinoma with squamous metaplasia | **Ⅱ** | 3.5×2.5×2cm | left lower lobe |
| adjacent tumor tissue/Primary lung adenocarcinoma | E05A2163 | lung | female | —— | chest wall and mediastinal  pleurolymph nodes | lung adenocarcinoma | **Ⅱ-Ⅲ** | 5*3.5*3.5cm | right upper lobe |
| adjacent tumor tissue/Primary lung adenocarcinoma | E05A0196 | lung | female | 65 | diaphragm | lung adenocarcinoma | **Ⅱ** | 5×4×3cm | right lower lobe |
| adjacent tumor tissue/Primary lung adenocarcinoma | E05A0952 | lung | male | 69 | rib | lung adenocarcinoma | **Ⅱ-Ⅲ** | 3.5×2.5×2cm | left upper lobe |
| adjacent tumor tissue/Primary lung adenocarcinoma | E05A0997 | lung | male | 57 | brain | lung adenocarcinoma | **Ⅱ** | 4×3×2cm | right upper lobe |
| Metastasis of lung adenocarcinoma | P01A0132 | brain | female | 45 | brain | Metastasis of lung adenocarcinoma |  |  | left frontal lobe |
| Metastasis of lung adenocarcinoma | P01A0135 | brain | female | 58 | brain | Metastasis of lung adenocarcinoma |  |  | brain |
| Metastasis of lung adenocarcinoma | P01A0099 | brain | female | 65 | brain | Metastasis of lung adenocarcinoma |  |  | right temporal |
| Metastasis of lung adenocarcinoma | E06A0018 | Soft tissue of chest wall | male | 65 | Soft tissue of chest wall | Metastasis of lung adenocarcinoma |  |  | left anterior chest wall |
| negative lymph node | E05A0243 | lung | male | 59 |  | lung adenocarcinoma | | 10*7*6cm | left upper lobe |
| negative lymph node | E05A0325 | lung | male | 71 |  | lung adenocarcinoma | | 5.5*5*4cm | left lower lobe |
| negative lymph node | E05A0326 | lung | male | 50 |  | lung adenocarcinoma | | 5*5*5cm | left lower lobe |
| negative lymph node | E05A0378 | lung | female | 54 |  | lung adenocarcinoma | | 3*3*2.5cm | right middle lobe |
| negative lymph node | E05A0379 | lung | female | 62 |  | lung adenocarcinoma | | 2.5*2*2cm | left upper lobe |
| negative lymph node | E05A0397 | lung | male | 74 |  | lung adenocarcinoma | | 3.5*3*3cm | right upper lobe |
| negative lymph node | E05A0599 | lung | male | 65 |  | lung adenocarcinoma | | 5*4*3cm | right lung |
| positive lymph node | E05A0174 | lung | female | 50 |  | lung adenocarcinoma | | 3*2.5*2cm | right lower lobe |
| positive lymph node | E05A0260 | lung | female | 56 |  | lung adenocarcinoma | | diameter 5cm | left lower lobe |
| positive lymph node | E05A0324 | lung | male | 72 |  | lung adenocarcinoma | | 3.5*3*3cm | left upper lobe |
| positive lymph node | E05A0507 | lung | female | 61 |  | lung adenocarcinoma | | 2*1.5*1cm | left upper lobe |
| positive lymph node | E05A0731 | lung | male | 50 |  | lung adenocarcinoma | | 3*3*2.8cm | right upper lobe |
| positive lymph node | E05A0783 | lung | female | 56 |  | lung adenocarcinoma | | 5*5*3cm | left upper lobe |

**Table S3. Reagents and manufacturers.**

| **Antibodies and reagents** | **Manufacturer** | **Cat number** | **Dilution ratio for IHC** | **Dilution ratio for IF** | **Dilution ratio for WB** |
| --- | --- | --- | --- | --- | --- |
| anti-Profilin1 | Abcam | ab124904 | 1:500 | 1:100 | 1:2500 |
| anti-p-MYL12A | Abclonal | AP0412 | 1:100 |  | 1:1000 |
| anti-Annexin A1 | Servicebio | GB14007 |  | 1:100 |  |
| anti-ROCK1 | Cell Signaling Technology | 4035S |  |  | 1:1000 |
| anti-ROCK2 | Cell Signaling Technology | 9029S |  |  | 1:1000 |
| anti-ERK | Cell Signaling Technology | 4348S |  |  | 1:1000 |
| anti-p-ERK | Cell Signaling Technology | 4370S |  |  | 1:1000 |
| anti-E-cad | Abcam | ab76055 |  |  | 1:1000 |
| anti-ARF6 | Immunoway | YT0309 |  |  | 1:1000 |
| anti-β-actin | Antgene | ANT321 |  |  | 1:5000 |
| anti-MYL12A | Abclonal | A9176 |  |  | 1:1000 |
| anti-N-cad | Cell Signaling Technology | 13116S |  |  | 1:1000 |
| anti-Vimentin | Abcam | ab92547 |  |  | 1:2500 |
| anti-HA | MBL | M180-3 |  |  | 1:5000 |
| anti-MYC | MBL | M192-3 |  |  | 1:5000 |
| anti-GAPDH | Antgene | ANT324 |  |  | 1:5000 |
| anti-DDDK | Antgene | ANT301 |  |  | 1:5000 |
| HRP Goat anti Mouse IgG(H+L) | Antgene | ANT019 |  |  | 1:5000 |
| HRP Goat anti Rabbit IgG(H+L) | Antgene | ANT020 |  |  | 1:5000 |
| 3% bovine serum albumin | Servicebio | .G5001 |  |  |  |
| VECTASTAIN Elite ABC HRP Kit | VECTOR LABORATISE | PK6100 |  |  |  |
| VECTOR DAB kit | VECTOR LABORATISE | SK-4100 |  |  |  |
| 3%BSA | Servicebio | G5001 |  |  |  |
| DAPI | Servicebio | G1012 |  |  |  |
| ClonExpress II One Step Cloning Kit | Vazyme Biotech Co. | C115-01 |  |  |  |
| 2 × Phanta Master Mix | Vazyme Biotech Co. | P511-01 |  |  |  |
| FastPure Plasmid Mini Kit | Vazyme Biotech Co. | DC201, |  |  |  |
| Lipofectamine™ 3000 Transfection Reagent | Invitrogen | L3000015 | |  |  |
| 1% crystal violet | Solarbio | G1062 |  |  |  |
| PKH67 | Sigma | MIDI67-1KT | |  |  |
| PrimeScript RT reagent Kit | Takara | RR047A |  |  |  |
| Mir-X-miRNA First-Strand Synthesis Kit | Takara | 638314 |  |  |  |
| TB Green Premix qPCR Mix | Takara | RR820A |  |  |  |
| protease inhibitor | Beyotime | P0013B |  |  |  |
| cocktail | Bimake | B14002 |  |  |  |
| Pierce™ Protein A/G Magnetic Beads | Thermofisher Scientific | 88802 |  |  |  |
| ROCK Kinase assay | Abcam | ab211175 | |  |  |

**Table S4. Primer sequences.**

| **Primers** | **Sequence (5' to 3')** |  |
| --- | --- | --- |
| PFN1 Primer F | GTTCGTCAACATCACGCCAG | |
| PFN1 Primer R | GTCCCGGATCACCGAACATT | |
| GAPDH Primer F | TCGGAGTCAACGGATTTGGT | |
| GAPDH Primer R | TGGAATTTGCCAT GGGTGGA | |
| Plvx-PFN1 WT vector Primer F | TTCCTCGAGACTAGTTCTAGAATGGCCGGTGGAACGCCT | |
| Plvx-PFN1 WT vector Primer R | GGAGGGAGAGGGGCGGGATCCTCAGTACTGGGAACGCCGAAG | |
| Plvx-PFN1 H119E vector Primer F | AGAAGGTGTCGAAGGTGGTTTGATCAACAAGAAA | |
| Plvx-PFN1 H119E vector Primer R | TCAAACCACCTTCGACACCTTCTTTGCCCATCAG | |
| Plvx-PFN1 H133S vector Primer F | AATGGCCTCCTCCCTTCGGCGTTCCCAGTACTGA | |
| Plvx-PFN1 H133S vector Primer R | ACGCCGAAGGGAGGAGGCCATTTCATAACATTTC | |
| Plvx-PFN1 R88L vector Primer F | ATGGATCTTCTTACCAAGAGCACCGGTGGGGCC | |
| Plvx-PFN1 R88L vector Primer R | GCTCTTGGTAAGAAGATCCATGCTAAATTCCCCA | |
| PFN1 siRNA-1 | ACC ACC GUG GAC ACC UUC UUU | |
| PFN1 siRNA-2 | GCA AAG ACC GGU CAA GUU U | |
