# Supplementary material for: Profilin 1 Induces Tumor Metastasis by Promoting Microvesicle Secretion Through the ROCK 1/p-MLC Pathway in Non-Small Cell Lung Cancer
Source: Front Pharmacol. 2022 May 2;13:890891. doi: 10.3389/fphar.2022.890891 (PMC9108340; doi:10.3389/fphar.2022.890891)
Supplement: Supplementary file 2 [file DataSheet1.docx]

***Supplementary Data***

**Supplementary figures and figure legends**

***
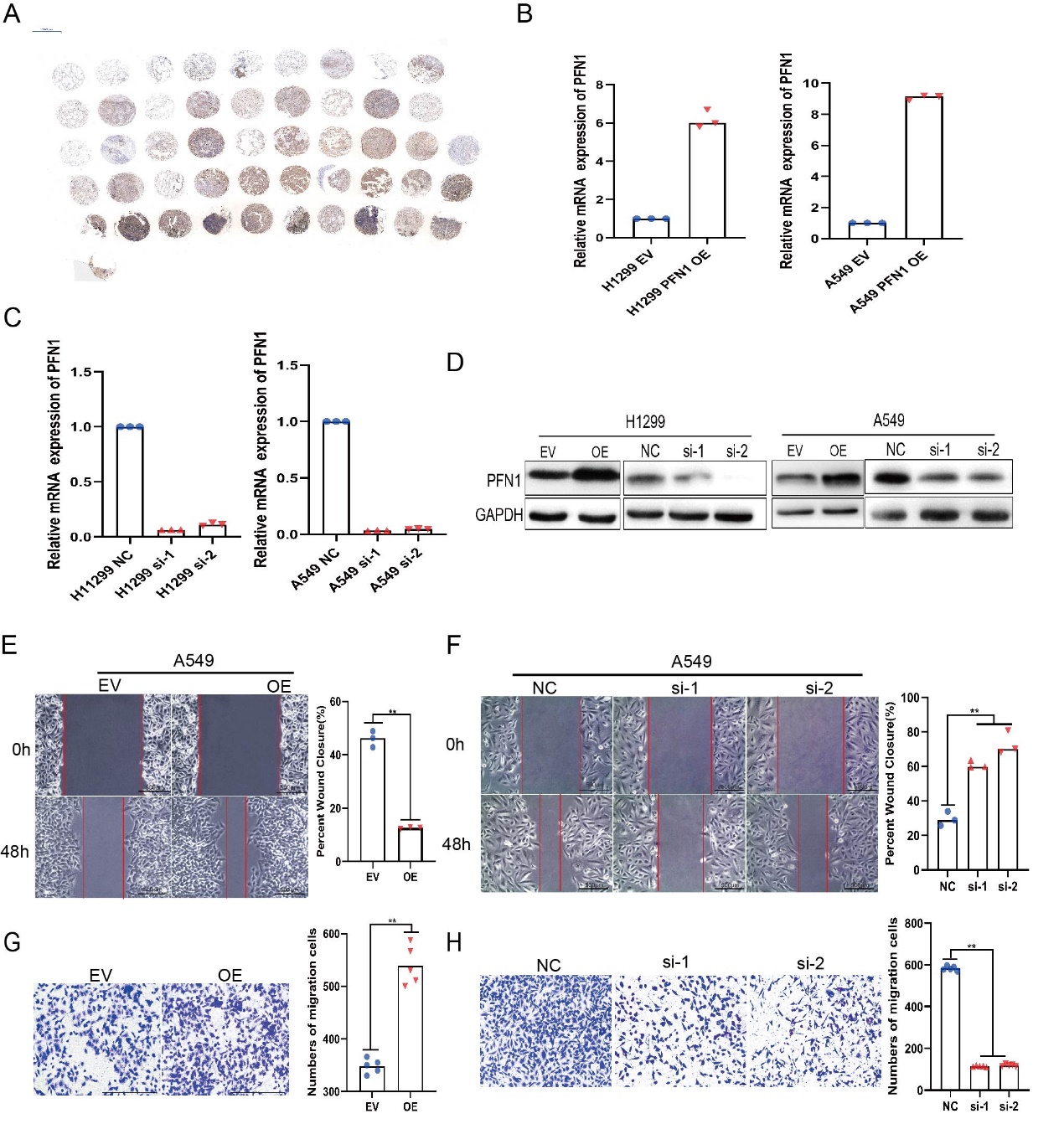
***

**Supplementary Figure S1. PFN1’s roles in NSCLC metastasis.**

**(A)** Scanned pictures of immunohistochemistry (IHC) analysis of PFN1 expression on the tissue chip. **(B)** and **(C)** RT-qPCR conducted to detect *PFN1* expression after infection of cells with lentivirus (B) or treatment with siRNA (C). **(D)** PFN1 protein level assessed using western blotting after transfection with PFN1 overexpression plasmids and siRNA. **(E)** and **(F)** Wound healing assays conducted to evaluate the migration ability of *PFN1*-overexpressing (E) and *PFN1* knockdown (KD) (F) A549 cells. **p < 0.01; scale bar, 500 μm. **(G)** and **(H)** Transwell migration assays conducted to evaluate the migration of *PFN1*-overexpressing (G) and *PFN1* KD (H) A549 cells. **p < 0.01; scale bar, 500 μm. EV: empty vector; OE: *PFN1* overexpression; NC: negative control; si-1/ 2: PFN1 siRNA1 1/2.


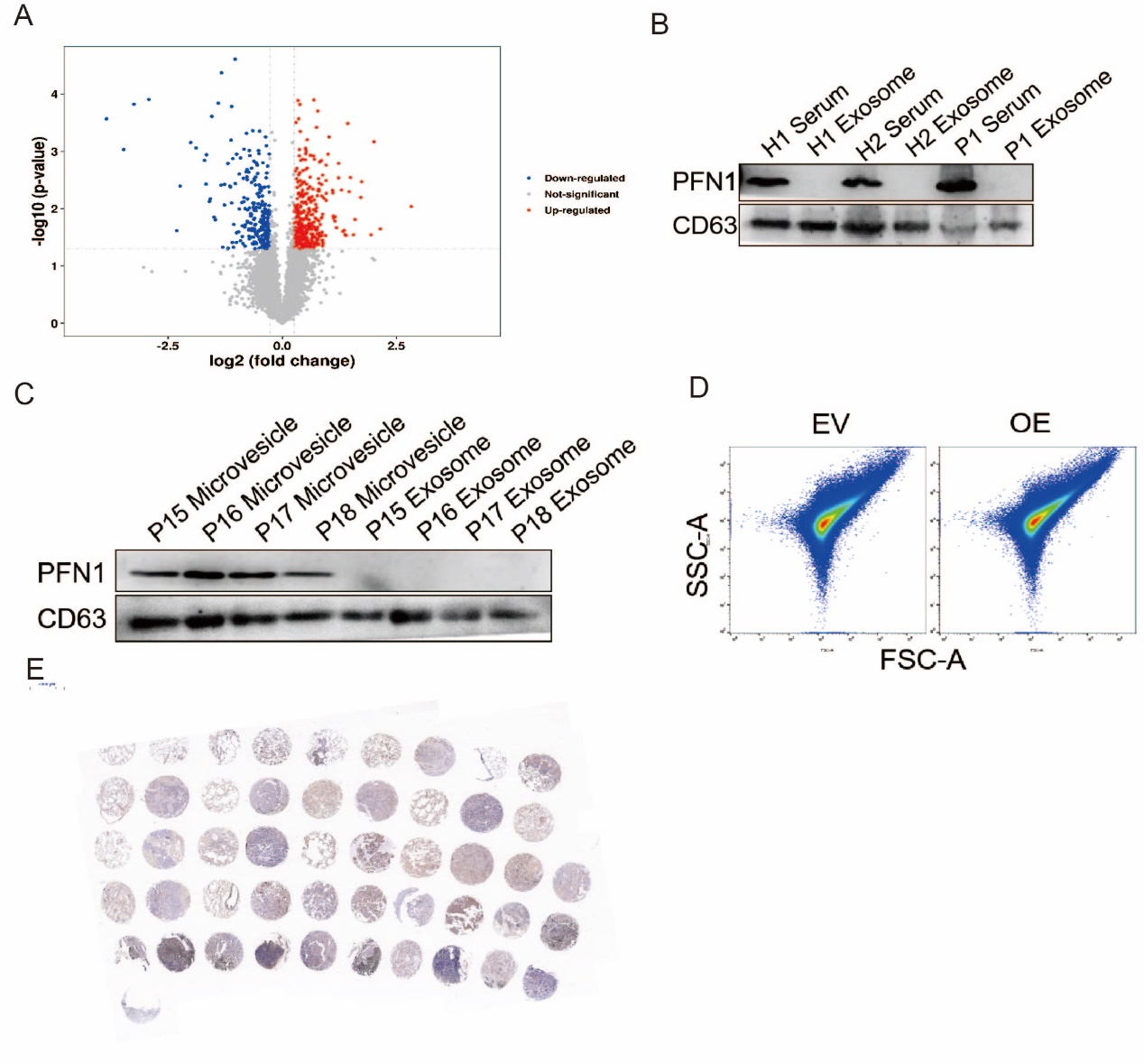


**Supplementary Figure S2. The correlation of PFN1 and MVs secretion.**

**(A)** Volcano plot of differentially expressed proteins. **(B)** Protein levels of PFN1 and CD63 in the sera of patients and exosomes extracted from sera. **(C)** Protein levels of PFN1 and CD63 in microvesicles and exosomes extracted from sera of patients. H: healthy donor; P: NSCLC patient. **(D)** Representative images of flow cytometry of MVs. **(E)** Scanned pictures of IHC analysis of p-MLC expression on the tissue chip.


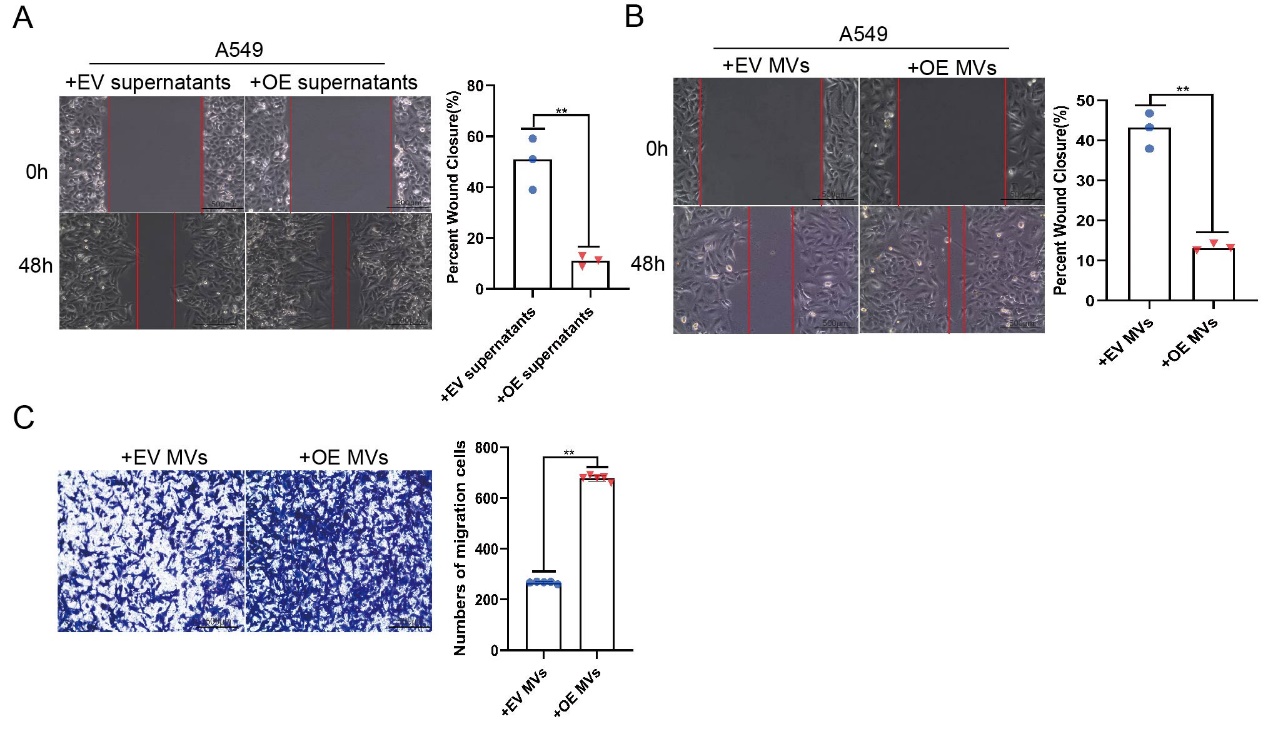


**Supplementary Figure S3. MVs promote A549 cells migration.**

**(A),** Effect of *PFN1*-overexpressing cell supernatants on cell migration evaluated through wound healing assays. **p < 0.01; scale bar, 500 μm. **(B)** and **(C)** Wound healing (B) and Transwell migration (C) assays conducted to evaluate the migration of H1299 cells after treatment with MVs derived from EV-expressing and *PFN1*-overexpressing cells. **p < 0.01; scale bar, 500 μm.

**
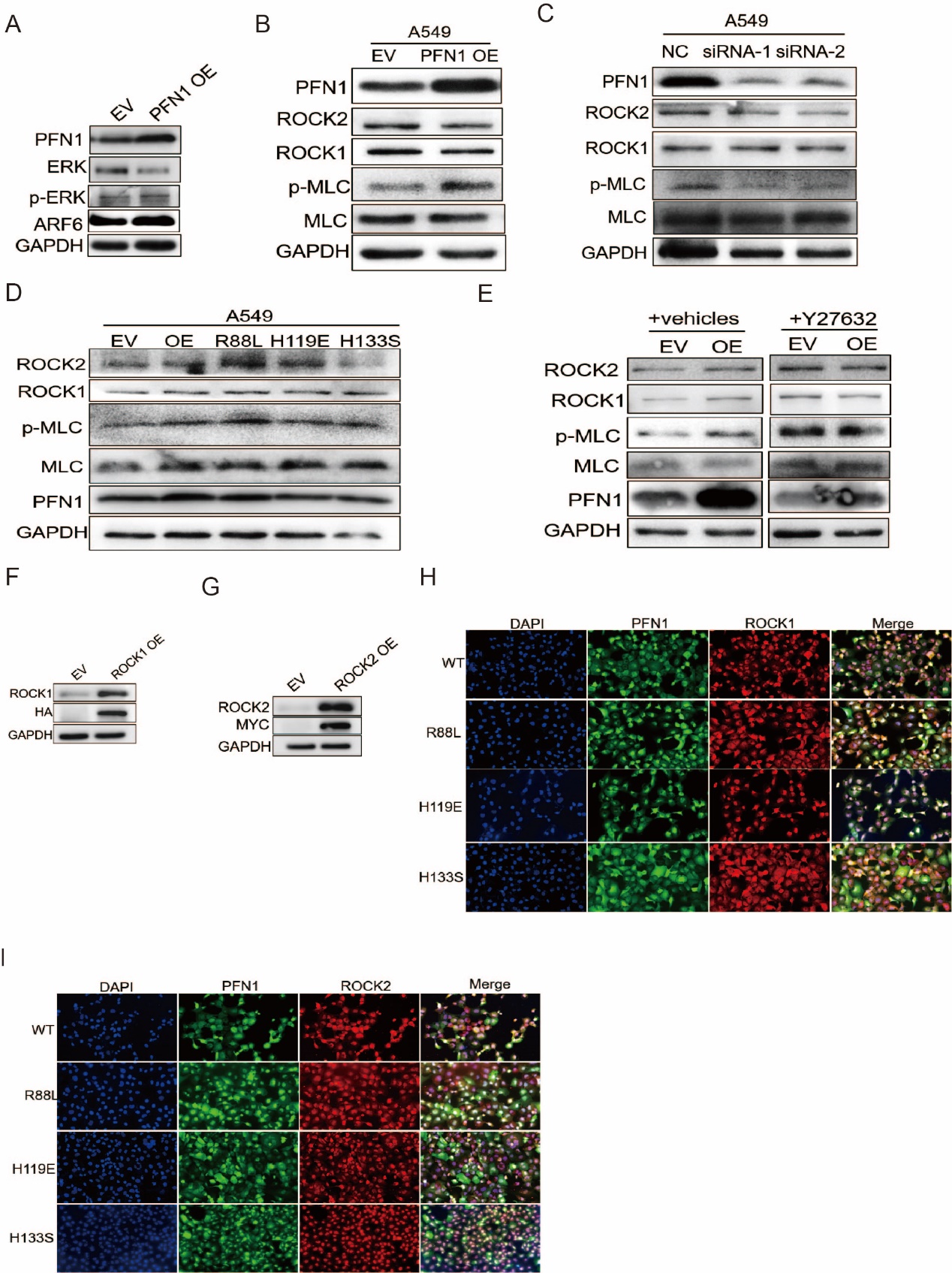
**

**Supplementary Figure S4.** **Mechanisms underlying the promotion of myosin light chain (MLC) phosphorylation by PFN1.**

**(A)** Expression of ERK, p-ERK, and ARF6 in *PFN1*-overexpressing cells detected using western blotting. **(B)** and **(C)**ROCK1/2, MLC, p-MLC and PFN1 expression in *PFN1*-overexpressing (B) and *PFN1* KD A549 cells (C) detected using western blotting. (**D)** ROCK1/2, p-MLC, MLC, and PFN1 expression in PFN1 mutant-expressing A549 cells. **(E)** Protein expression after treatment with Y27632 (10 µM) measured using western blotting. **(F)** and **(G)** Transfection efficiency of ROCK1 (F) and ROCK2 (G) plasmids detected using western blotting. (**H)** and (**I**) GFP tagged PFN1 and its mutants together with immunofluorescence targeting ROCK1 (H) and ROCK2 (I).


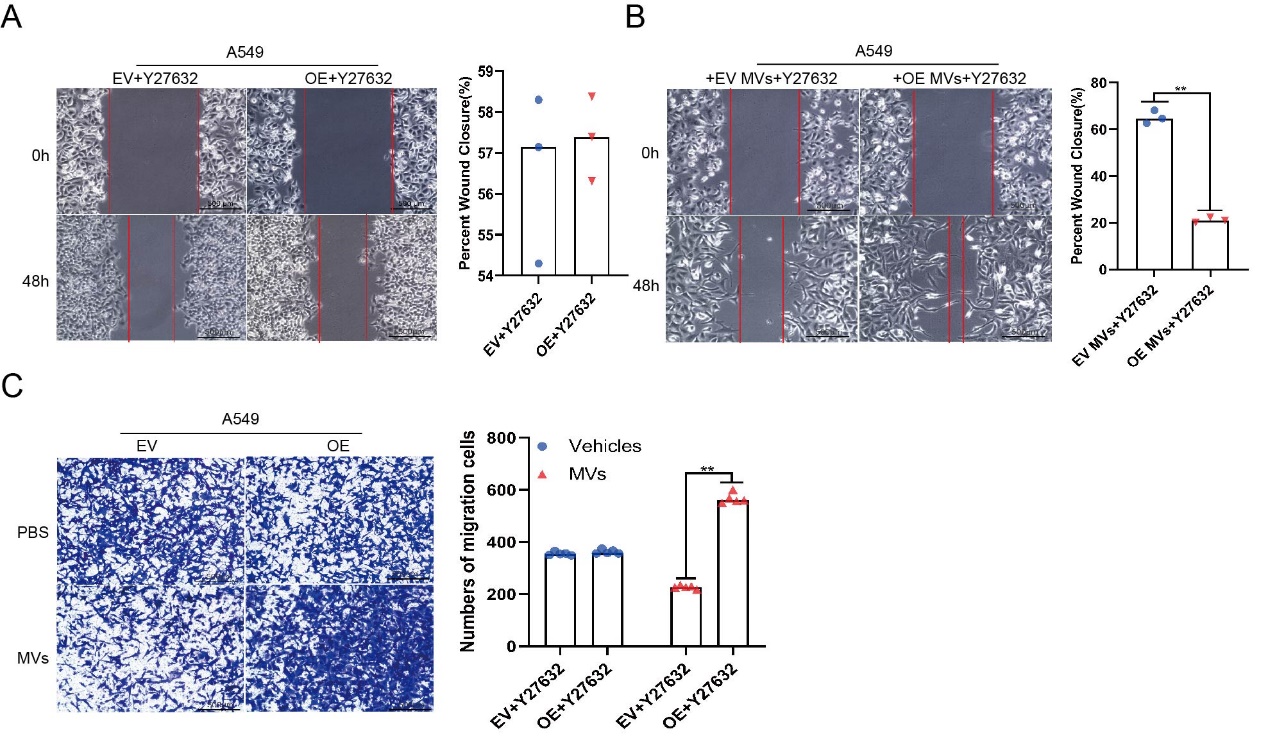


**Supplementary Figure S5. ROCK1 inhibitor Y27632 partially reversed PFN1-induced A549 cell migration in vitro.**

**(A)** and **(B)** Wound healing assays conducted to evaluate the effect of Y27632 (A) and Y27632 combined with microvesicles (MVs) (B) on cell migration. **p < 0.01; scale bar, 500 μm. (**C)** Transwell migration assays conducted to evaluate the effect of Y27632 and Y27632 combined with MVs on cell migration. **p < 0.01; scale bar, 500 μm.
